# Supplementary material for: Repeated information of benefits reduces COVID-19 vaccination hesitancy: Experimental evidence from Germany
Source: PLoS One. 2022 Jun 28;17(6):e0270666. doi: 10.1371/journal.pone.0270666 (PMC9239477; doi:10.1371/journal.pone.0270666)
Supplement: S1 Appendix — (PDF) [file pone.0270666.s001.pdf]

S1 Appendix. Survey registration, and pre-test

Pre-registration

Data collection and analysis were preregistered on AsPredicted (Ref. 66735) and can be accessed at https://aspredicted.org/WAA_TNE. The aim of our study is to investigate whether information and reminders can encourage participants to get vaccinated as measured by their self-reported intention to vaccinate and their vaccination action.

We outlined our plan to conduct three surveys between Mai and December 2021. At the time of writing, the third and final survey has not yet been conducted. However, as the last survey was intended only as a follow-up in the event of low vaccination rates or supply shortages in the summer – neither of which has occurred – we are confident that the results of the first two surveys are already informative and important.

We intended to capture participants' other-regarding preferences by using the 6-item social value orientation task (SVO) from [1]. However, due to a programming error, we were unable to reliably capture participants' responses, resulting in several missing responses. Therefore, we decided to exclude the social value orientation task from our analysis. In S9 Appendix we show that this does not affect our main results.

Pre-test

We pre-tested our survey experiment with 575 students from the University of Marburg in May 2021. Thanks to the often very detailed comments we received, we were able to improve and clarify some questions. This also allowed us to arrange the information in the debunking and benefit treatment so that the information rated "most interesting" in the pretest was displayed first.

We also used the pre-test to reduce [2] dogmatism scale from 18 items to 10 items. There were two reasons for this: We wanted to keep the questionnaire as short as possible for the participants, and

some of the items seemed repetitive, especially after translation into German. For example, item number 18, see Table S1, was excluded because it seemed to cause confusion among participants and we, therefore, considered the responses unreliable. We apply principal component analysis to exclude items with low loadings. In the first iteration, we excluded all items with loadings below .19. This threshold was set to avoid prematurely excluding items that were close to .20. In the second iteration, in which the loadings for the remaining items are recalculated, we exclude all items with loadings below .20. Further reduction of items would have reduced the explanatory power of the principal component analysis. For reasons of comparability and reproducibility, we report average values in our analysis to control for participants' dogmatic attitudes, not principal components.

Table S1. *Complete dogmatism scale*

Variable	*To what extent do you agree with the following statements?*<br>*[Do not agree at all 1 |---| 7 Fully agree]*	***Preserved***
dt06_01	1. Anyone who is honestly and truly seeking the truth will end up believing what I believe.	X
dt06_02	2. There are so many things we have not discovered yet, nobody should be absolutely certain his beliefs are right. R	
dt06_03	3. The things I believe in are so completely true, I could never doubt them.	X
dt06_04	4. I have never discovered a system of beliefs that explains everything to my satisfaction. R	
dt06_05	5. It is best to be open to all possibilities and ready to reevaluate all your beliefs. R	
dt06_06	6. My opinions are right and will stand the test of time.	X
dt06_07	7. Flexibility is a real virtue in thinking, since you may well be wrong. R	
dt06_08	8. My opinions and beliefs fit together perfectly to make a crystal-clear "picture" of things.	X
dt06_09	9. There are no discoveries or facts that could possibly make me change my mind about the things that matter most in life.	X
dt06_10	10. I am a long way from reaching final conclusions about the central issues in life. R	x
dt06_11	11. The person who is absolutely certain she has the truth will probably never find it. R	
dt06_12	12. I am absolutely certain that my ideas about the fundamental issues in life are correct.	X
dt06_13	13. I am so sure I am right about the important things in life, there is no evidence that could convince me otherwise.	X
dt06_14	14. If you are "open-minded" about the most important things in life, you will probably reach the wrong conclusions.	
Dt06_15	15. Twenty years from now, some of my opinions about the important things in life will probably have changed. R	x
dt06_16	16. "Flexibility in thinking" is another name for being "wishsy-washy".	X
dt06_17	17. No one knows all the essential truths about the central issues in life. R	
dt06_18	18. Someday I will probably realize my present ideas about the BIG issues are wrong. R	

Note: Table S1 shows the complete dogmatism scale from Altemeyer (2002) and what items were included in our final measurement.

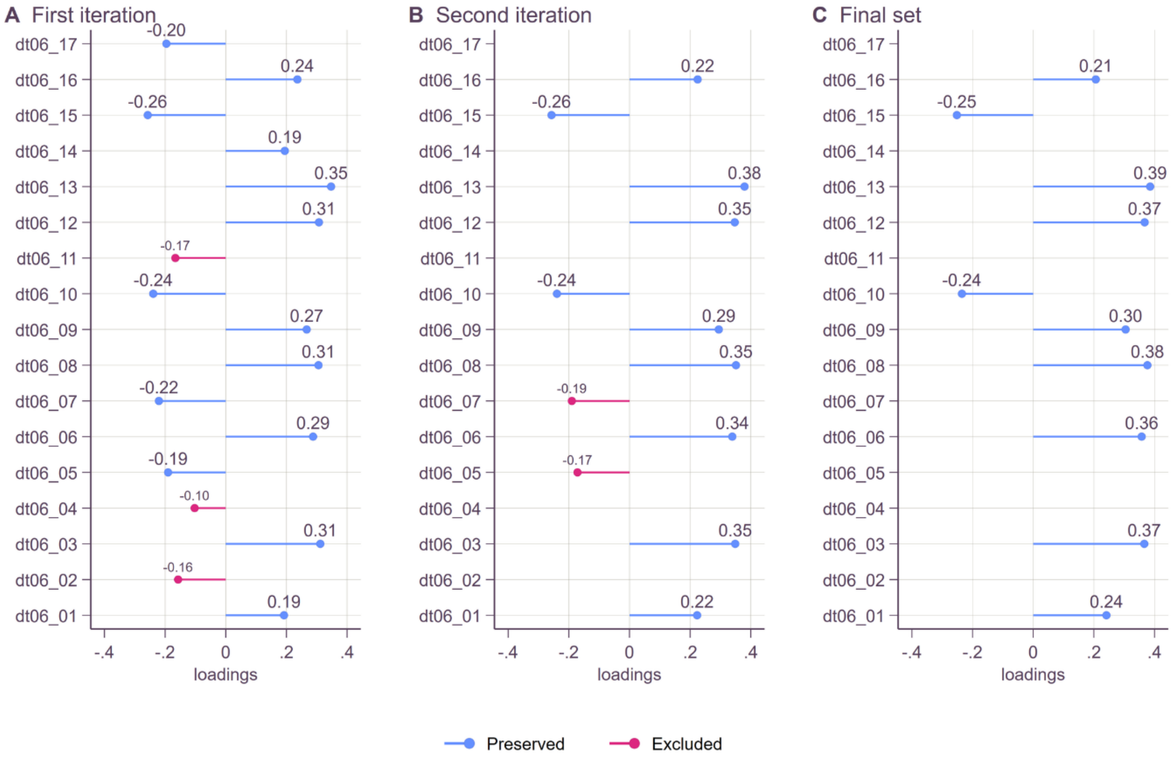

Fig S1. Compression of dogmatism scale. Loadings of the individual items from [2] complete dogmatism scale, see Table S1, are shown. Items that were excluded are highlighted in orange. Using principal component analysis, we calculate the loadings for each item. Panel A shows the first iteration, in which all items with loadings below .19 were excluded. Panel B shows the second iteration, where all items below .20 were excluded. Panel C shows the loadings of the remaining items.

References

1. Murphy J, Vallières F, Bentall RP, Shevlin M, McBride O, Hartman TK, et al. Psychological characteristics associated with COVID-19 vaccine hesitancy and resistance in Ireland and the United Kingdom. Nat Commun. 2021;12: 29. doi:10.1038/s41467-020-20226-9

2. Altemeyer B. Dogmatic behavior among students: Testing a new measure of dogmatism. J Soc Psychol. 2002;142: 713–721. doi:10.1080/00224540209603931
