# Supplementary material for: Repeated information of benefits reduces COVID-19 vaccination hesitancy: Experimental evidence from Germany
Source: PLoS One. 2022 Jun 28;17(6):e0270666. doi: 10.1371/journal.pone.0270666 (PMC9239477; doi:10.1371/journal.pone.0270666)
Supplement: S2 Appendix — (PDF) [file pone.0270666.s002.pdf]

S2 Appendix. Literature review

In this section, we briefly review the literature on vaccine hesitancy in general and hesitancy towards COVID-19 vaccines in particular. As we considered this review too extensive for the main manuscript, we decided to include only a brief summary in the introduction and present the rest here.

Vaccination hesitancy in general

Vaccination hesitancy – the reluctance or refusal to vaccinate despite the availability of vaccines – has existed ever since the first vaccines were developed in the early 1800s and has never completely gone away since then [1,2]. While the methods of information-sharing and motives of the anti-vaccination movements have changed over the last 200 years, a great overlap in arguments remains. Vaccines continue to be portrayed as ineffective, disease-causing, containing harmful substances, and authorities are accused to conceal the harm caused by vaccines. Moreover, it is argued that alternative health products such as homeopathy or vitamins are superior to vaccines and that natural immunity is better than vaccine-induced immunity. Thus, vaccination mandates are presented as a violation of civil rights and only serve the companies that manufacture vaccines for profit [1]. The growth of the anti-vaccine movement poses an ever-increasing problem for global health, as more and more people view vaccines as unsafe and unnecessary [3,4]. At the beginning of the 2000s vaccination rates plummeted across many high-income countries [2,5–7] as well as low- and middle-income countries [8]. This led to a "comeback" of vaccine-preventable diseases that were once brought under control – such as measles, poliomyelitis, and pertussis [9,10].

Decades of research on vaccination hesitancy have shown that hesitancy is complex, multifaceted, and context-specific (for an overview see [11]). A large share of variation in hesitancy can be explained by differences in (i) sociodemographic characteristics, (ii) cultural, institutional, and political factors, as well as (iii) psychological factors [5,6,8,10,12,13]. However, there is not one

universal factor that consistently explains hesitancy as determinants vary not only across countries and vaccine types but also over time. While sociodemographic characteristics – such as age, gender, socio-economic status, ethnicity – are the most reported determinants of vaccination hesitancy literature reviews found large inconsistencies in these variables. Some studies associate higher socioeconomic status and age with higher procrastination while others find the opposite effect [12,13]. Furthermore, norms such as encouragement from others or social pressure, as well as knowledge on the vaccine and vaccine-preventable diseases increased uptake in some countries [12,13]. But health knowledge influenced by myths and rumors (Nigeria) as well as anthroposophist beliefs (Netherlands) worked as a barrier [12].

One approach to mapping the different determinants of vaccination hesitancy is the 5C model, which elicits people's Confidence in the safety and efficacy of the vaccine, Complacency about the risk of infection, Constraints that prevent one from vaccinating, Calculation of one's own costs and benefits, and the perceived Collective responsibility to vaccinate [14]. This approach is based on extensive literature review and testing by the Strategic Advisory Group of Experts on Immunization (SAGE) who worked out three key determinants of vaccination hesitancy [15]: The trust in the effectiveness and safety of the vaccine as well as health workers and policymakers (Confidence), the belief of the need of the vaccine (Complacency), and the ability to obtain the vaccination (Complacency). This model was later extended to include people's perceived costs and benefits of vaccination (Calculation) [16] and by the willingness to protect others (Collective responsibility) to the 5C model [14]. While there is some discussion in the literature to include further categories [17], we decided to adopt the more established 5C model in our study.

Vaccination hesitancy towards COVID-19 vaccines

The development of the COVID-19 vaccines provided a unique opportunity to study vaccination hesitancy on an unprecedented scale. Vaccine development was extensively covered by the media around the world and vaccines were released simultaneously in many countries, although

predominantly in high-income countries first. Furthermore, COVID-19 vaccines were relevant to all segments of society, in contrast to many previous vaccination campaigns that primarily focused on parents vaccinating their children [5,12]. Since most studies on COVID-19 vaccination hesitancy that have been published at the time of writing investigate hesitancy based on vaccination intentions, not actions, conclusion on what drives this hesitancy might change over time as more studies are published that also consider vaccination actions.

Nevertheless, the research on COVID-19 vaccines hesitancy already sheds some light on the connection of (i) sociodemographic characteristics, (ii) cultural, institutional, and political factors, as well as (iii) psychological factors that might explain hesitancy. Sociodemographic characteristics such as age, gender, affluence, and education seem to be important for vaccination hesitancy across many different contexts [18,19]. While many studies in western countries find that female, younger, less educated, and less affluent individuals are more likely to be hesitant in Australia [20], France [21], the US [22–24], as well as Ireland and the UK [25], there are some notable exceptions. In Denmark, for example, females seem to be more supportive of COVID-19 vaccination than males [24]. Lazarus et al. [26] surveying 13,426 people in 19 countries conclude that globally speaking being male, older, less educated, less affluent is associated with higher hesitancy.

Cultural, political, and institutional differences such as lower trust in authorities [19,20,25,27,28] and support of populist views [20,29] were also found to correlate with higher COVID-19 hesitancy. It seems that COVID-19 policies, as well as the vaccination debate, were politicized in many countries. It is therefore not surprising that studies find a correlation between vaccination hesitancy and support for a particular party, such as the republicans in the US [23], or the far-right AfD in Germany [30]. There are also likely to be institutional factors – as indicated by the varying levels of trust in authorities – and cultural factors – such as the prevalence of anthroposophical movements [31]. These factors are certainly important to explain how hesitancy emerges, yet it is difficult, sometimes even impossible, to change them on short notice. Therefore, we focus more on

psychological determinants here, as interventions that target people's attitudes, perceptions, and opinions are likely more fruitful in swiftly reducing hesitancy.

Ruiz and Bell [23], for example, find that general vaccination knowledge and personal COVID-19 threat appraisal are associated with higher vaccination acceptance in the US; Murphy et al. [25] find that – among other psychological determinants – lower levels of trust in scientists, health care professionals, and the state, as well as lower levels of altruism, are associated with higher hesitancy in both Ireland and the UK; and Edwards et al. [20] find that people with greater confidence in their government, hospitals, and state systems were less likely to be against vaccination in Australia. Furthermore, Gerretsen et al [29] show that two of the three factors of the 3Cs – Confidence (38%) and Complacency (21%) – model have the highest explanatory power before socio-demographics (13%) and other psychological factors (11%) in the US and Canada. While Wismans et al. [17] applying the 5C model find that Confidence and Collective Responsibility are most strongly related to COVID-19 vaccination hesitancy in a survey including 1,137 university students from the Netherlands, Belgium, and Portugal. Applying mediation analyses, the authors argue that "the perceived risk and effectiveness of the vaccine as well as trust in the government and health authorities indirectly relate to vaccination intention through Confidence." Furthermore, "the perceived risk of COVID-19 for one's social circle and altruism, the need to belong and psychopathy traits indirectly relate to vaccination intention through Collective Responsibility."

Vaccination hesitancy in Germany

Studies looking at vaccine hesitancy in Germany prior to COVID-19 find that vaccination hesitancy correlates with lower risk appraisal of the disease, negative attitudes towards immunization, or having a migration background [32–34]. Nevertheless, hesitancy seems to decrease if physicians recommend vaccination [32] or simply when having a family physician [33]. Interestingly, vaccination hesitancy prior to COVID-19 was also found to be lower for persons residing in parts of former East Germany [32–34]. For COVID-19 vaccines this correlation is reversed, with higher

hesitancy in the east compared to the west [30]. Beyond regional differences COVID-19 vaccination hesitancy seems to correlate with being female, older, better educated, and more affluent in Germany [35–37]. However, COVID-19-related anxiety, fears of infection, overall risk appraisal of the pandemic, and support for COVID-19 policies appear to be strong indicators for getting vaccinated [35–37].

Overall, hesitancy in Germany is likely related to the prevalent tradition of Anthroposophy [31], widespread acceptance of homeopathy funded by the health care system [38], and support for right-wing politics [30]. In these aspects, Germany seems to be comparable to other German-speaking countries – Austria and Switzerland – with whom it shares substantial cultural, historical, and economic ties. All three countries have a similar system of federalism, operate a system of mandatory universal health care, and applied similar strategies to contain the initial COVID-19 outbreak – school closures, the obligatory wearing of masks, social distancing, increased testing, and contact tracing. Shared cultural elements, such as low intergenerational contact and cohabitation, may also have contributed to keeping initial fatalities low [39]. A less positive development these three countries share is the plateauing of vaccination rates at a suboptimal level (between 50 and 65%) by the end of September 2021 the reason for which is seen in a high vaccination hesitancy [30]. Although the reasons for this hesitation are admittedly complex and interrelated, some similarities between the German-speaking countries can be identified. In all three countries vaccination rates vary regionally – Germany: Bremen 78% vs Saxony 56%, Austria: Burgenland 67% vs. Oberösterreich 55%, Switzerland: Ticino 57% vs 42% Appenzell Innerrhoden). Furthermore, support for right-wing parties (Germany: Alternative for Germany (AfD), Austria: Freedom Party of Austria (FPO), Switzerland: Swiss People’s Party (SVP)) has been found to be associated with higher vaccination hesitancy [30].

References

1. Dubé E, Vivion M, MacDonald NE. Vaccine hesitancy, vaccine refusal and the anti-vaccine movement: influence, impact and implications. Expert Rev Vaccines. 2015;14: 99–117. doi:10.1586/14760584.2015.964212

2. Wolfe RM, Sharp LK. Anti-vaccinationists past and present. 2002;325: 3.

3. Dubé È, Ward JK, Verger P, MacDonald NE. Vaccine Hesitancy, Acceptance, and Anti-Vaccination: Trends and Future Prospects for Public Health. Annu Rev Public Health. 2021;42: 175–191. doi:10.1146/annurev-publhealth-090419-102240

4. de Figueiredo A, Simas C, Karafillakis E, Paterson P, Larson HJ. Mapping global trends in vaccine confidence and investigating barriers to vaccine uptake: a large-scale retrospective temporal modelling study. The Lancet. 2020;396: 898–908. doi:10.1016/S0140-6736(20)31558-0

5. Dubé E, Laberge C, Guay M, Bramadat P, Roy R, Bettinger JA. Vaccine hesitancy: An overview. Hum Vaccines Immunother. 2013;9: 1763–1773. doi:10.4161/hv.24657

6. Williams SE. What are the factors that contribute to parental vaccine-hesitancy and what can we do about it? Hum Vaccines Immunother. 2014;10: 2584–2596. doi:10.4161/hv.28596

7. McIntosh EDG, Janda J, Ehrich JHH, Pettoello-Mantovani M, Somekh E. Vaccine Hesitancy and Refusal. J Pediatr. 2016;175: 248-249.e1. doi:10.1016/j.jpeds.2016.06.006

8. Rainey JJ, Watkins M, Ryman TK, Sandhu P, Bo A, Banerjee K. Reasons related to non-vaccination and under-vaccination of children in low and middle income countries: Findings from a systematic review of the published literature, 1999–2009. Vaccine. 2011;29: 8215–8221. doi:10.1016/j.vaccine.2011.08.096

9. Paules CI, Marston HD, Fauci AS. Measles in 2019 — Going Backward. N Engl J Med. 2019;380: 2185–2187. doi:10.1056/NEJMp1905099

10. Falagas ME, Zarkadoulia E. Factors associated with suboptimal compliance to vaccinations in children in developed countries: a systematic review. Curr Med Res Opin. 2008;24: 1719–1741. doi:10.1185/03007990802085692

11. Dubé E, Gagnon D, MacDonald NE. Strategies intended to address vaccine hesitancy: Review of published reviews. Vaccine. 2015;33: 4191–4203. doi:10.1016/j.vaccine.2015.04.041

12. Larson HJ, Jarrett C, Eckersberger E, Smith DMD, Paterson P. Understanding vaccine hesitancy around vaccines and vaccination from a global perspective: A systematic review of published literature, 2007–2012. Vaccine. 2014;32: 2150–2159. doi:10.1016/j.vaccine.2014.01.081

13. Schmid P, Rauber D, Betsch C, Lidolt G, Denker M-L. Barriers of Influenza Vaccination Intention and Behavior – A Systematic Review of Influenza Vaccine Hesitancy, 2005 – 2016. Cowling BJ, editor. PLOS ONE. 2017;12: e0170550. doi:10.1371/journal.pone.0170550

14. Betsch C, Schmid P, Heinemeier D, Korn L, Holtmann C, Böhm R. Beyond confidence: Development of a measure assessing the 5C psychological antecedents of vaccination. PLOS ONE. 2018;13: e0208601. doi:10.1371/journal.pone.0208601

15. Strategic Advisory Group of Experts on Immunization. Report of the SAGE working group on vaccine hesitancy. World Health Organization; 2014 Oct.

16. Betsch C, Böhm R, Chapman GB. Using Behavioral Insights to Increase Vaccination Policy Effectiveness. Policy Insights Behav Brain Sci. 2015;2: 61–73. doi:10.1177/2372732215600716

17. Wismans A, Thurik R, Baptista R, Dejardin M, Janssen F, Franken I. Psychological characteristics and the mediating role of the 5C Model in explaining students' COVID-19 vaccination intention. Delcea C, editor. PLOS ONE. 2021;16: e0255382. doi:10.1371/journal.pone.0255382

18. Troiano G, Nardi A. Vaccine hesitancy in the era of COVID-19. Public Health. 2021;194: 245–251. doi:10.1016/j.puhe.2021.02.025

19. Cascini F, Pantovic A, Al-Ajlouni Y, Failla G, Ricciardi W. Attitudes, acceptance and hesitancy among the general population worldwide to receive the COVID-19 vaccines and their contributing factors: A systematic review. EClinicalMedicine. 2021;40: 101113. doi:10.1016/j.eclinm.2021.101113

20. Edwards B, Biddle N, Gray M, Sollis K. COVID-19 vaccine hesitancy and resistance: Correlates in a nationally representative longitudinal survey of the Australian population. Di Gennaro F, editor. PLOS ONE. 2021;16: e0248892. doi:10.1371/journal.pone.0248892

21. Schwarzinger M, Watson V, Arwidson P, Alla F, Luchini S. COVID-19 vaccine hesitancy in a representative working-age population in France: a survey experiment based on vaccine characteristics. Lancet Public Health. 2021;6: e210–e221. doi:10.1016/S2468-2667(21)00012-8

22. Daly M, Robinson E. Willingness to Vaccinate Against COVID-19 in the U.S.: Representative Longitudinal Evidence From April to October 2020. Am J Prev Med. 2021;60: 766–773. doi:10.1016/j.amepre.2021.01.008

23. Ruiz JB, Bell RA. Predictors of intention to vaccinate against COVID-19: Results of a nationwide survey. Vaccine. 2021;39: 1080–1086. doi:10.1016/j.vaccine.2021.01.010

24. Petersen MB, Bor A, Jørgensen F, Lindholt MF. Transparent communication about negative features of COVID-19 vaccines decreases acceptance but increases trust. Proc Natl Acad Sci. 2021;118. doi:10.1073/pnas.2024597118

25. Murphy J, Vallières F, Bentall RP, Shevlin M, McBride O, Hartman TK, et al. Psychological characteristics associated with COVID-19 vaccine hesitancy and resistance in Ireland and the United Kingdom. Nat Commun. 2021;12: 29. doi:10.1038/s41467-020-20226-9

26. Lazarus JV, Ratzan SC, Palayew A, Gostin LO, Larson HJ, Rabin K, et al. A global survey of potential acceptance of a COVID-19 vaccine. Nat Med. 2021;27: 225–228. doi:10.1038/s41591-020-1124-9

27. Lindholt MF, Jørgensen F, Bor A, Petersen MB. Public acceptance of COVID-19 vaccines: cross-national evidence on levels and individual-level predictors using observational data. BMJ Open. 2021;11: e048172. doi:10.1136/bmjopen-2020-048172

28. Kerr JR, Freeman ALJ, Marteau TM, van der Linden S. Effect of Information about COVID-19 Vaccine Effectiveness and Side Effects on Behavioural Intentions: Two Online Experiments. Vaccines. 2021;9: 379. doi:10.3390/vaccines9040379

29. Gerretsen P, Kim J, Caravaggio F, Quilty L, Sanches M, Wells S, et al. Individual determinants of COVID-19 vaccine hesitancy. Inbaraj LR, editor. PLOS ONE. 2021;16: e0258462. doi:10.1371/journal.pone.0258462

30. Desson Z, Kauer L, Otten T, Peters JW, Paolucci F. Finding the way forward: COVID-19 vaccination progress in Germany, Austria and Switzerland. Health Policy Technol. 2021; 100584. doi:10.1016/j.hlpt.2021.100584

31. Fournet N, Mollema L, Ruijs WL, Harmsen IA, Keck F, Durand JY, et al. Under-vaccinated groups in Europe and their beliefs, attitudes and reasons for non-vaccination; two systematic reviews. BMC Public Health. 2018;18: 196. doi:10.1186/s12889-018-5103-8

32. Boes L, Boedeker B, Schmich P, Wetzstein M, Wichmann O, Remschmidt C. Factors associated with parental acceptance of seasonal influenza vaccination for their children – A telephone survey in the adult population in Germany. Vaccine. 2017;35: 3789–3796. doi:10.1016/j.vaccine.2017.05.015

33. Rehmet S, Ammon A, Pfaff G, Bocter N, Petersen LR. Cross-Sectional Study on Influenza Vaccination, Germany, 1999–2000. Emerg Infect Dis. 2002;8: 1442–1447. doi:10.3201/eid0812.010497

34. Poethko-Müller C, Ellert U, Kuhnert R, Neuhauser H, Schlaud M, Schenk L. Vaccination coverage against measles in German-born and foreign-born children and identification of unvaccinated subgroups in Germany. Vaccine. 2009;27: 2563–2569. doi:10.1016/j.vaccine.2009.02.009

35. Graeber D, Schmidt-Petri C, Schröder C. Attitudes on voluntary and mandatory vaccination against COVID-19: Evidence from Germany. Capraro V, editor. PLOS ONE. 2021;16: e0248372. doi:10.1371/journal.pone.0248372

36. Umakanthan S, Lawrence S. Predictors of COVID-19 vaccine hesitancy in Germany: a cross-sectional, population-based study. Postgrad Med J. 2022; postgradmedj-2021-141365. doi:10.1136/postgradmedj-2021-141365

37. Bendau A, Plag J, Petzold MB, Ströhle A. COVID-19 vaccine hesitancy and related fears and anxiety. Int Immunopharmacol. 2021;97: 107724. doi:10.1016/j.intimp.2021.107724

38. Altenbuchner A, Haug S, Schnell R, Scharf A, Weber K. Impfbereitschaft von Eltern mit einem COVID-19-Vakzin: Die Rolle von Elternschaft und Geschlecht. Pädiatr Pädologie. 2021;56: 230–234. doi:10.1007/s00608-021-00925-2

39. Desson Z, Lambertz L, Peters JW, Falkenbach M, Kauer L. Europe’s Covid-19 outliers: German, Austrian and Swiss policy responses during the early stages of the 2020 pandemic. Health Policy Technol. 2020;9: 405–418. doi:10.1016/j.hlpt.2020.09.003
