# Supplementary material for: Repeated information of benefits reduces COVID-19 vaccination hesitancy: Experimental evidence from Germany
Source: PLoS One. 2022 Jun 28;17(6):e0270666. doi: 10.1371/journal.pone.0270666 (PMC9239477; doi:10.1371/journal.pone.0270666)
Supplement: S3 Appendix — (PDF) [file pone.0270666.s003.pdf]

S3 Appendix. Data collection and study site background

While Germany never faced a hard lockdown in which freedom of movement was completely restricted, several other policies were implemented to reduce the spread of COVID-19. In Mai 2021, contact restrictions in private and public spaces were still in place. Moreover, anyone traveling to a high incidence area had to go into quarantine for 14 days or until they received a negative test result. When COVID-19 vaccines became towards the end of 2020, the German government put a vaccination prioritization system in place: Highest priority: Being above 80, living in care facilities being highly vulnerable, or working in care facilities being highly exposed. High priority: Being above 70, certain illnesses making people vulnerable, up to two contact persons of each person needing care, and working in areas of mid exposition. Increased priority: Being above 60, having certain illnesses, working with low exposition, and working in areas of high importance (alimentation, transport, pharmacy, etc.). Vaccination prioritization was officially lifted in Germany on June 5th. Yet, since appointments were organized several weeks in advance, people were already invited to sign up for appointments mid May 2021. As we were interested in participants intention to get vaccinated and their subsequent actions, we chose this period were vaccinations were made widely available as the first survey phase. Hence, the survey experiment was implemented between Mai 25th and June 2nd. In the following months, with much of the demand satisfied waiting lists and sometimes even appointments became obsolete in vaccination centers. As a substantial part of the population still was unvaccinated despite availability of vaccines, policies were discussed to increase the vaccination uptake. At the time of the study, monetary incentives have been discussed but not employed. Mobile vaccination teams, invitations for vaccination, and vaccination in public places (e.g., in front of sport stadiums at game-days) have been put to practice between the survey experiment and the follow-up survey. Exclusion from public events for unprotected (unvaccinated and not recovered) and stop of free official testing were not employed throughout the study period. However, after our study was completed, free official testing was stopped for about one month

between October 11 and November 13. Furthermore, access to stores (except food retailers, pharmacies, post office, etc.) and restaurants was restricted to those vaccinated or recovered in several federated states in Germany from the beginning of December onwards, i.e., both stop free testing and exclusion of non-vaccinated or non-cured were already a topic of debate during the study period. At the time of our follow up survey between September 6th and 18th 2021 all participants have had ample opportunity to be vaccinated. Furthermore, as all policies tested in our survey have been debated in the general public at that time it is likely that they have thought about the measures themselves beforehand. For example, to counteract the spread of vaccination myths, the German government (https://www.zusammengegencorona.de/) and RKI (https://www.rki.de/SharedDocs/FAQ/COVID-Impfen/gesamt.html) set up pages providing corrective information, such as in Switzerland (https://bag-coronavirus.ch/impfung/nebenwirkungen-fragen/) or the US (https://www.cdc.gov/vaccines/COVID-19/health-departments/addressing-vaccine-misinformation.html).
