# Supplementary material for: Repeated information of benefits reduces COVID-19 vaccination hesitancy: Experimental evidence from Germany
Source: PLoS One. 2022 Jun 28;17(6):e0270666. doi: 10.1371/journal.pone.0270666 (PMC9239477; doi:10.1371/journal.pone.0270666)
Supplement: S4 Appendix — (PDF) [file pone.0270666.s004.pdf]

S4 Appendix. Exclusion criteria

This section outlines which observations were not included in the analysis.

Survey experiment

As we outlined during pre-registration, we invited only unvaccinated participants. Nevertheless, 1.2% (91 out of 1,623) of participants who completed the survey reported being either partially or fully vaccinated at the first survey in May 2021. These individuals were therefore excluded from the study. As outlined in the pre-registration, participants who completed the survey in less than 5 minutes (4.5%, 69 out of 1,532) or showed signs of inattention (1%, 16 out of 1,463) were excluded.

Additionally, at the end of the survey, we asked participants if they answered all questions as instructed, if they were unable to answer certain questions due to technical issues, or if they gave random answers. Participants were informed that their answer would not affect their payment, as everyone who successfully completed the survey would receive payment. We did not include this exclusion criterion in the pre-registration because we were unsure how well it would work. Of the 1,447 participants, 4 (.3%) reported having technical issues, 77 (5.3%) reported occasionally have given random answers, and 7 (.5%) participants reported frequently giving random responses. To improve the overall quality of the data, we believe it is better to exclude data where participants report having given random responses. Therefore, we decided to exclude these observations from the main analysis.

Finally, having a panel dataset allows us to check if participants provided accurate responses by controlling for whether their reported age changed by more than one year (2.2%, 30 out of 1,359) or whether participants changed gender between the two surveys in May and September 2021 (.4%, 5 out of 1,329). Excluding these observations yields a final sample of N = 1,324 observations for the survey experiment. While participants excluded differ from those not excluded (see Table S2)

the overall results do not change by excluding participants in the analysis of the survey experiment (Table S13) and the panel (Table S16).

Table S2. *Balance table: Main sample and excluded*

Variable	(1) Main sample N	(1) Main sample Mean/SE	(2) Excluded N	(2) Excluded Mean/SE	Difference t-test (1)-(2)
Control variables					
Female (=1)	1324	0.511	208	0.385	0.127***
		[0.014]		[0.034]	
Age: <30	1324	0.202	208	0.308	-0.105***
		[0.011]		[0.032]	
Age: 30-39	1324	0.128	208	0.231	-0.103***
		[0.009]		[0.029]	
Age: 40-49	1324	0.218	208	0.149	0.069**
		[0.011]		[0.025]	
Age: 50-64	1324	0.371	208	0.240	0.130***
		[0.013]		[0.030]	
Age: 65+	1324	0.081	208	0.072	0.009
		[0.007]		[0.018]	
Secondary school:	1324	0.113	207	0.164	-0.051**
"Hauptschulabschluss"		[0.009]		[0.026]	
Secondary school:	1324	0.347	207	0.329	0.018
"Realschulabschluss"		[0.013]		[0.033]	
High school:	1324	0.284	207	0.266	0.018
"Fach & allg. Hochschulberechtigung"		[0.012]		[0.031]	
University degree	1324	0.256	207	0.242	0.014
		[0.012]		[0.030]	
Adjusted Household-Income	1324	4.013	208	3.552	0.460***
		[0.049]		[0.124]	
Married	1324	0.412	208	0.370	0.041
		[0.014]		[0.034]	
Explanatory variables					
Denied other vaccines	1324	0.141	208	0.163	-0.022
		[0.010]		[0.026]	
Index: COVID-19 risk perception	1324	39.409	208	41.704	-2.295
		[0.617]		[1.335]	
Index: Emotional response to COVID-19	1324	3.612	208	3.678	-0.066
		[0.041]		[0.094]	
Net anticipated regret	1324	0.951	208	0.188	0.763***
(no vaccination – vaccination)		[0.110]		[0.220]	
Index: Dogmatism	1324	3.954	208	4.165	-0.210***
		[0.026]		[0.054]	
Outcome variables					
Vaccination intention: mRNA	1324	5.166	208	4.567	0.599***
		[0.064]		[0.147]	
Vaccination intention: Vector	1324	3.319	208	3.462	-0.143
		[0.064]		[0.139]	
5C: Confidence mRNA	1324	4.709	208	4.457	0.252*
		[0.054]		[0.121]	
5C: Confidence Vector	1324	3.909	208	3.944	-0.035
		[0.051]		[0.110]	
5C: Constraints	1324	2.174	208	3.207	-1.033***
		[0.037]		[0.115]	
5C: Complacency	1324	3.187	208	3.882	-0.695***
		[0.049]		[0.114]	
5C: Calculation	1324	5.080	208	4.918	0.162
		[0.044]		[0.095]	

5C: Collective responsibility	1324	5.052	208	4.556	0.496***
		[0.050]		[0.102]	
Vaccination inaction (=1)	1324	0.525	208	0.510	0.015
		[0.014]		[0.035]	

Note: The value displayed for t-tests are the differences in the means across the groups. ***, **, and * indicate significance at the 1, 5, and 10 percent critical level. Column (1) shows the means of the reduced sample used for the analysis in the paper. Column (2) shows the means for those participants excluded from the analysis due to the criteria defined. One participant excluded (column 2) specified his education in a way that could not be attributed to the abovementioned categories.

Balanced panel

In September 2021, 987 participants completed the second survey. Applying the same criteria as in the first survey, 30 out of 987 (3.0%) participants were excluded because they completed the survey in less than 5 minutes, but no observations were removed due to lack of attention. Of the remaining 957 participants, 2 (.2%) reported experiencing technical issues, 58 reported occasionally giving random responses, and 6 reported frequently giving random responses. Removing these observations yields a sample size of N = 891 of which we further exclude 37 (4.2%) participants who were excluded from the first survey due to the exclusion criteria. Finally, we also remove the 28 participants who changed their age by more than one year and the 5 participants who changed gender, resulting in a final sample size of N = 821 for the balanced panel.
