# Supplementary material for: Repeated information of benefits reduces COVID-19 vaccination hesitancy: Experimental evidence from Germany
Source: PLoS One. 2022 Jun 28;17(6):e0270666. doi: 10.1371/journal.pone.0270666 (PMC9239477; doi:10.1371/journal.pone.0270666)
Supplement: S5 Appendix — (PDF) [file pone.0270666.s005.pdf]

S5 Appendix. Attrition

As expected not all participants who took part in the survey experiment in May 2021 returned for the follow-up survey in September 2021. Although 987 of 1,324 participants completed the follow-up survey, only 821 observations are considered for analysis due to the pre-registered exclusion criteria, resulting in an overall attrition rate of 38%. Attrition could pose a risk to the internal validity of our treatment effects on vaccination inaction. In the following, we aim to identify whether there is a problem and, if so, the size of it. As a first step, we determine whether attrition rates are different across treatment and control groups. Fig. S2 shows that attrition differs across groups. While there are no significant differences between attrition rates of the control group and both information treatments, participants in the facilitation group are about 6 percentage points less likely to return (T-Test diff. = -.062, t_{669} = -1.63, p = .103). While this does not necessarily threaten internal validity, it is more likely that participants in the facilitation treatment differ in baseline outcomes or important determinants of the outcome variable from the control group.

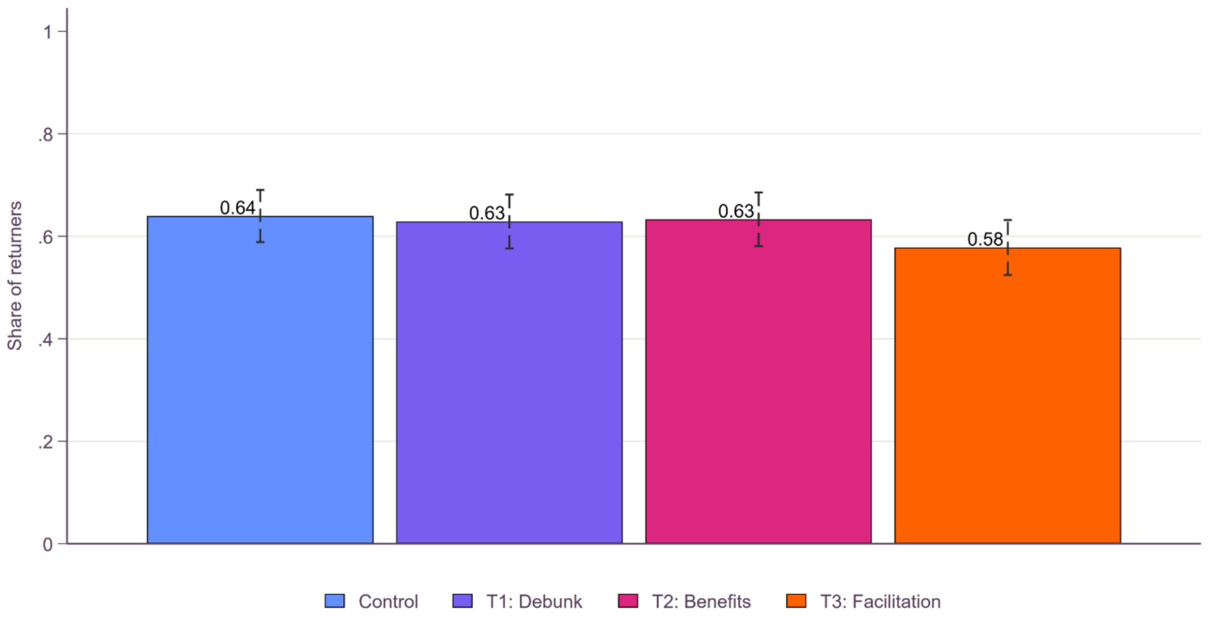

Fig S2. Differential attrition across treatments. Shares by treatment group and 95% confidence intervals Plotted are the shares of participants who took part in both the survey experiment and follow-up survey for each treatment group.

In a second step, we analyze whether there is selective attrition, i.e., whether the mean of baseline outcomes differ between treatment groups for attritors and returners. Random assignment in the presence of attrition does not ensure that any differences between control and treatment groups can be attributed to the treatment. These comparisons are only unbiased if we were able to collect follow-up data for all participants from the survey experiment. To understand whether attrition affects the internal validity of our estimates, we compare the mean baseline outcomes across all four groups of participants by return status [1], see Table S3. For baseline outcomes (inaction, intention, 5C), we find that returners (panel A) and attritors (panel B) have similar values across all four groups. Only returners in the debunking treatment have significantly lower values for the Calculation dimension of the 5C scale than control returners. On average across all groups, however, there are some significant differences between returners and attritors, for example, baseline inaction is about 50% for attritors and 54% for returners. Also, attritors tend to have higher vaccination intentions than returners. This indicates that returning to the follow-up survey is correlated with other characteristics unobserved in our survey (or maybe even unobservable) that affect our outcome of interest. However, these differences are still independent of the treatment assignment within returners and within attritors. Thus, while our returner sample might not be representative of the study population from the survey experiment, our average treatment estimates are still internally valid for the returner population. While differences between treatments for attritors are not significant for our outcomes, there are not zero. For example, baseline inaction is 46% for control attritors but 56% for information treatment attritors. Due to these differences, we rather overestimate the effectiveness of our information treatments. To account for these imbalances, we control for interaction effects with baseline inaction in our analysis reported in S9 Appendix.

Table S3. *Selective attrition across treatments*

	Mean Baseline Outcomes by Group				Differences in means		
	Control<br>(1)	T1: Debunk<br>(2)	T2: Benefits<br>(3)	T3: Facilitation<br>(4)	(1)-(2)	(1)-(3)	(1)-(4)
Panel A. Returners							
Vaccination inaction	0.532	0.522	0.517	0.582	0.010	0.015	-0.050
Vaccination intention: mRNA	4.936	5.073	5.309	4.884	-0.137	-0.373	0.053
5C: Confidence mRNA	4.574	4.691	4.805	4.450	-0.117	-0.231	0.125
5C: Complacency	3.307	3.073	3.097	3.249	0.234	0.210	0.058
5C: Constraints	2.035	1.997	2.196	2.071	0.038	-0.162	-0.036
5C: Calculation	5.323	4.928	5.143	5.339	0.394***	0.179	-0.016
5C: Collective responsibility	5.005	4.920	5.116	4.921	0.084	-0.111	0.084
Observations	220	205	207	189			
Panel B. Attritors							
Vaccination inaction	0.460	0.562	0.558	0.449	-0.102	-0.099	0.010
Vaccination intention: mRNA	5.387	5.273	5.233	5.493	0.114	0.154	-0.106
5C: Confidence mRNA	4.645	4.890	4.783	4.998	-0.245	-0.138	-0.352
5C: Complacency	3.274	3.186	3.371	2.978	0.088	-0.097	0.296
5C: Constraints	2.371	2.306	2.425	2.256	0.065	-0.054	0.115
5C: Calculation	4.777	4.895	5.008	4.966	-0.118	-0.231	-0.189
5C: Collective responsibility	5.070	5.088	5.067	5.348	-0.018	0.003	-0.278
Observations	124	121	122	138			

Notes: The value displayed for t-tests are the differences in the means across the groups. ***, **, and * indicate significance at the 1, 5, and 10 percent critical level.

Reference

1. Ghanem D, Hirshleifer S, Ortiz-Becerra K. Testing Attrition Bias in Field Experiments. 2021 [cited 7 Dec 2021]. doi:10.26085/C38C76
