# Supplementary material for: Repeated information of benefits reduces COVID-19 vaccination hesitancy: Experimental evidence from Germany
Source: PLoS One. 2022 Jun 28;17(6):e0270666. doi: 10.1371/journal.pone.0270666 (PMC9239477; doi:10.1371/journal.pone.0270666)
Supplement: S6 Appendix — (PDF) [file pone.0270666.s006.pdf]

S6 Appendix. Measurement details

Risks to self and others: Calculated score and adjustment

Risk perception was assessed in three steps. First, we asked participants a series of questions about the likelihood of infection, the severity of the disease, and long-term consequences, such as long COVID (α = .71). Based on these responses, we ranked their perception of risk to themselves and others on a scale from 0 “no risk” to 100 “high risk.” Participants were then asked to correct their ratings if they felt misrepresented by it (see Fig. S3 for adjustments made). Finally, the average of the two scores was taken to determine the risk perception score used in the analysis.

To elicit participants’ risk perception of COVID-19 we asked participants how likely they think it is that they/others will get infected and severe an infection would be for themselves and others (see S12 Appendix question 1-3). From these answers, we calculated an index (unweighted mean) for risk appraisal of personal risk and risk for others presented to the participant. They then had the opportunity to adjust the risk appraisal for personal risk and risk for others. This procedure was chosen as we first wanted to guide participants towards factors we deemed relevant for the evaluation of risk appraisal but also to give them the chance to adjust their scores in case they felt misrepresented by the items we chose. Most participants made either small or no adjustments at all, with only 20% and 17% making changes greater than 20 of the calculated index for the personal risk and risk for others respectively. The correlation between the calculated score and the adjusted score is 0,71 ($p < .01$) for risk appraisal for oneself, and .83 ($p < .01$) for others. Fig. S3 plots the calculated score against the adjusted score for personal risk (a) and risk for others (b).

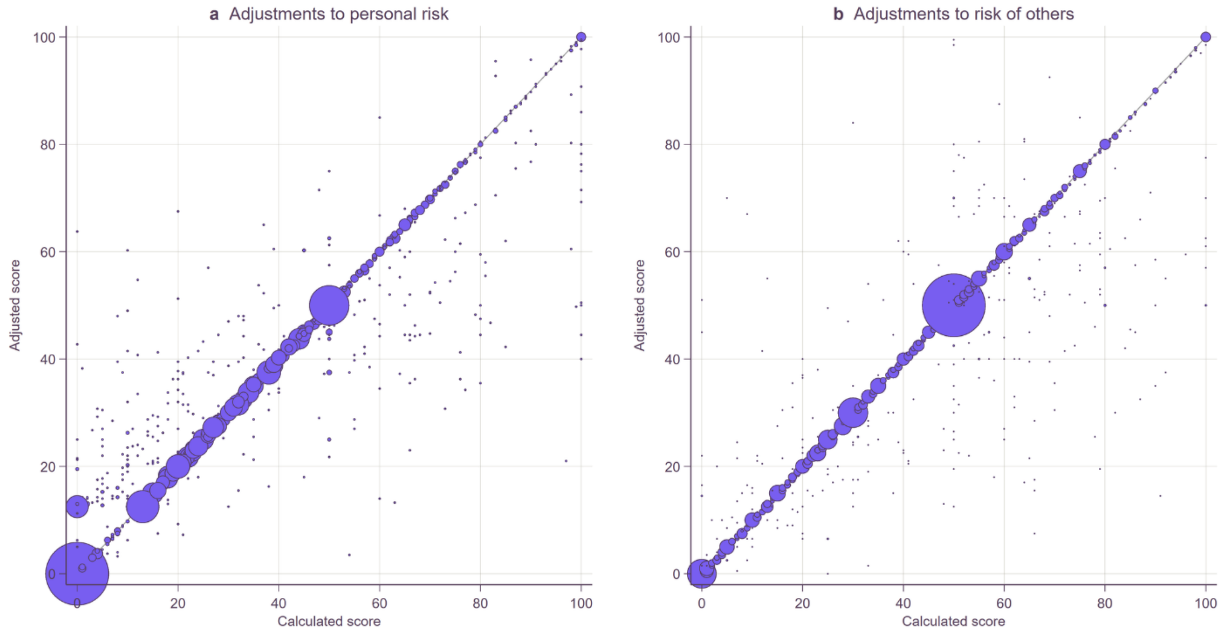

Fig S3. Participants' adjustments to the calculated risk perception score. Panel A shows the changes participants made to their personal risk perception score and panel B shows the adjustments made to participants' risk score for other people. Risk scores are calculated based on participants' responses. The calculated score that was shown to participants is plotted on the x-axis and the adjusted score on the y-axis. Observations that are to the left of the 45-degree line represent increases made by participants, and observations to the right represent decreases.

Need-Weighted net income approach

We follow the need-weighted net income approach (i.e., equivalent income) outlined by Niehues and Stockhausen [1] taking into account that children need less money than adults and that life becomes cheaper when several people live together. Therefore, the total monthly net household income is divided by the needs-weighted number of household members. The first adult has a factor of 1, each additional household member over 14 has a factor of .5, and children under 14 have a factor of .3.

Reference

1. Niehues J, Stockhausen M. Einkommensverteilung nach sozioökonomischen Teilgruppen. Niehues IW-Kurzber. 2019 [cited 8 Dec 2021]. Available: https://www.iwkoeln.de/studien/judith-niehues-maximilian-stockhausen-einkommensverteilung-nach-soziooekonomischen-teilgruppen-437725.html
