# Supplementary material for: Repeated information of benefits reduces COVID-19 vaccination hesitancy: Experimental evidence from Germany
Source: PLoS One. 2022 Jun 28;17(6):e0270666. doi: 10.1371/journal.pone.0270666 (PMC9239477; doi:10.1371/journal.pone.0270666)
Supplement: S7 Appendix — (PDF) [file pone.0270666.s007.pdf]

S7 Appendix. Balancing and manipulation check

Survey Experiment

Participants were assigned to treatment groups randomly. Despite random assignment differences between groups may occur. We test for this possibility to see for which variables we should control in the estimations. As we do find differences in age, education, past vaccine denial, risk perception, and emotional response to COVID-19 across treatments we control for these variables in all estimations.

Table S4. *Balancing across treatments: Survey experiment*

Variable	(1) Control Mean/SE	(2) Debunk Mean/SE	(3) Benefits Mean/SE	t-test Difference (1)-(2)	t-test Difference (1)-(3)
Socio-economics					
Female (=1)	0.520	0.503	0.502	0.017	0.019
	[0.019]	[0.028]	[0.028]		
Age: 32-44 (=1)	0.200	0.215	0.196	-0.015	0.004
	[0.015]	[0.023]	[0.022]		
Age: 32-44 (=1)	0.128	0.123	0.131	0.005	-0.003
	[0.013]	[0.018]	[0.019]		
Age: 45-52 (=1)	0.215	0.193	0.251	0.021	-0.036
	[0.016]	[0.022]	[0.024]		
Age: 53-58 (=1)	0.380	0.356	0.367	0.024	0.013
	[0.019]	[0.027]	[0.027]		
Age: 59-81 (=1)	0.077	0.113	0.055	-0.036*	0.022
	[0.010]	[0.018]	[0.013]		
Secondary school:	0.133	0.098	0.089	0.034	0.044**
‘Hauptschulabschluss' (=1)	[0.013]	[0.017]	[0.016]		
Secondary school: 'Realschuleabschluss'	0.343	0.377	0.324	-0.035	0.019
(=1)	[0.018]	[0.027]	[0.026]		
High school (=1)	0.273	0.282	0.309	-0.009	-0.036
	[0.017]	[0.025]	[0.026]		
University degree (=1)	0.252	0.242	0.278	0.010	-0.026
	[0.017]	[0.024]	[0.025]		
Adjusted HH income	3.951	4.098	4.054	-0.147	-0.102
	[0.069]	[0.100]	[0.100]		
Married (=1)	0.434	0.390	0.388	0.044	0.045
	[0.019]	[0.027]	[0.027]		
Explanatory variables					
Baseline: Vaccination inaction (=1)	0.516	0.537	0.532	-0.021	-0.016
	[0.019]	[0.028]	[0.028]		
Denied other vaccine (=1)	0.154	0.113	0.144	0.040*	0.010
	[0.014]	[0.018]	[0.019]		
Index: COVID-19 risk perception	41.171	37.523	37.674	3.648**	3.497**
	[0.900]	[1.202]	[1.171]		
Index: Emotional response	3.719	3.499	3.504	0.220**	0.215**
	[0.059]	[0.079]	[0.078]		
Net anticipated regret (no vaccination –	0.817	1.104	1.073	-0.288	-0.257
vaccination)	[0.151]	[0.227]	[0.225]		
Index: Dogmatism	3.934	4.002	3.948	-0.068	-0.014
	[0.037]	[0.053]	[0.053]		
Observations	671	326	327		
F-test of joint significance (F-stat)				1.804**	1.437
F-test, number of observations				997	998

Note: The value displayed for t-tests are the differences in the means across the groups. ***, **, and * indicate significance at the 1, 5, and 10 percent critical level.

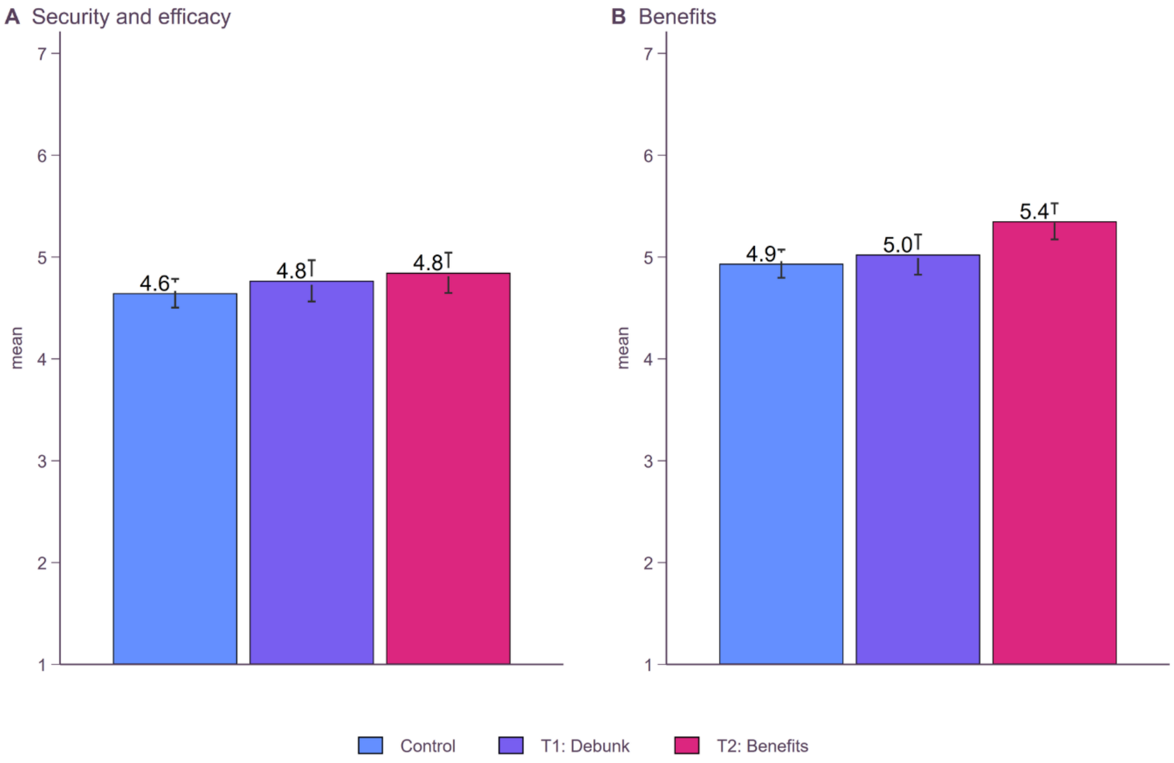

Fig S4. Priming check: Variation in feeling informed. Bars show mean values for each treatment group and 95% confidence intervals are indicated by lines. Panel A and B show the means towards the question "How informed do you feel about the safety and effectiveness of Corona vaccines?" and "How informed do you feel about the benefits for fully vaccinated individuals?" respectively. Answers were given on a 7-point Likert scale from 1 (not informed at all) to 7 (fully informed).

Balanced Panel

Table S5. *Balancing across treatments: Returners*

Variable	(1) Control Mean/SE	(2) T1: Debunk Mean/SE	(3) T2: Benefits Mean/SE	(4) T3: Facilitation Mean/SE	T-Test Differences (1)-(2)	(1)-(3)	(1)-(4)
Female (=1)	0.482	0.473	0.527	0.513	0.009	-0.045	-0.031
	[0.034]	[0.035]	[0.035]	[0.036]			
Age: 32-44 (=1)	0.105	0.146	0.140	0.090	-0.042	-0.036	0.015
	[0.021]	[0.025]	[0.024]	[0.021]			
Age: 32-44 (=1)	0.109	0.107	0.097	0.122	0.002	0.012	-0.013
	[0.021]	[0.022]	[0.021]	[0.024]			
Age: 45-52 (=1)	0.236	0.166	0.271	0.185	0.071*	-0.034	0.051
	[0.029]	[0.026]	[0.031]	[0.028]			
Age: 53-58 (=1)	0.432	0.449	0.430	0.513	-0.017	0.002	-0.081
	[0.033]	[0.035]	[0.034]	[0.036]			
Age: 59-81 (=1)	0.118	0.132	0.063	0.090	-0.014	0.055**	0.028
	[0.022]	[0.024]	[0.017]	[0.021]			
Secondary school:	0.127	0.112	0.077	0.169	0.015	0.050*	-0.042
'Hauptschulabschluss' (=1)	[0.023]	[0.022]	[0.019]	[0.027]			
Secondary school:	0.345	0.390	0.362	0.386	-0.045	-0.017	-0.041
'Realschuleabschluss' (=1)	[0.032]	[0.034]	[0.033]	[0.036]			
High school (=1)	0.250	0.254	0.290	0.233	-0.004	-0.040	0.017
	[0.029]	[0.030]	[0.032]	[0.031]			
University degree (=1)	0.277	0.244	0.271	0.212	0.033	0.007	0.066
	[0.030]	[0.030]	[0.031]	[0.030]			
Adjusted HH income	3.972	4.015	4.109	4.034	-0.042	-0.137	-0.062
	[0.120]	[0.119]	[0.120]	[0.137]			
Married (=1)	0.500	0.434	0.440	0.460	0.066	0.060	0.040
	[0.034]	[0.035]	[0.035]	[0.036]			
Baseline: Vaccination inaction (=1)	0.532	0.522	0.517	0.582	0.010	0.015	-0.050
	[0.034]	[0.035]	[0.035]	[0.036]			
Denied other vaccine (=1)	0.155	0.122	0.130	0.148	0.033	0.024	0.006
	[0.024]	[0.023]	[0.023]	[0.026]			
Index: COVID-19 risk perception	40.827	36.324	37.493	38.558	4.503*	3.335	2.269
	[1.645]	[1.592]	[1.456]	[1.712]			
Index: Emotional response	3.599	3.460	3.524	3.617	0.139	0.075	-0.018
	[0.109]	[0.104]	[0.101]	[0.114]			
Net anticipated regret (no	0.586	1.141	1.111	0.566	-0.555	-0.525	0.020
vaccination – vaccination)	[0.271]	[0.310]	[0.279]	[0.300]			
Index: Dogmatism	3.912	4.068	3.989	4.051	-0.157	-0.078	-0.140
	[0.066]	[0.072]	[0.067]	[0.072]			
Observations	220	205	207	189			
F-test of joint significance (F-stat)					1.329	1.336	0.869
F-test, number of observations					425	427	409

Notes: The dependent variable took the value of one when the participant was assigned to one of the treatments and zero when in control for each of the joint F-test of orthogonality.
