# Supplementary material for: Repeated information of benefits reduces COVID-19 vaccination hesitancy: Experimental evidence from Germany
Source: PLoS One. 2022 Jun 28;17(6):e0270666. doi: 10.1371/journal.pone.0270666 (PMC9239477; doi:10.1371/journal.pone.0270666)
Supplement: S8 Appendix — (PDF) [file pone.0270666.s008.pdf]

S8 Appendix. Sample details

Participants were recruited from a general population panel in Germany by the survey company Respondi, which has mainly access to Germans between the age of 18-69 years. We aimed at a gender and age distribution similar to that of the German population within the accessible age bracket from 18-69. For targeting, we used the age distribution of the German population provided by the Statistische Bundesamt. We formed six age categories (18-25, 26-35, 36-45, 46-55, 55-69, 70, and above) for male and female participants respectively. Recruitment for a given age-gender combination was stopped once the target aimed for was reached.

In terms of gender, our sample is not statistically different from the age distribution in Germany in the age between 18 and 70. However, our final sample exceeds the German distribution in terms of people between 55 and 60 and we fall below the German distribution for those above 60. This is most likely to be explained by the fact, that we only allowed unvaccinated people to participate in a time when older people were more likely to be vaccinated due to vaccination priorities.

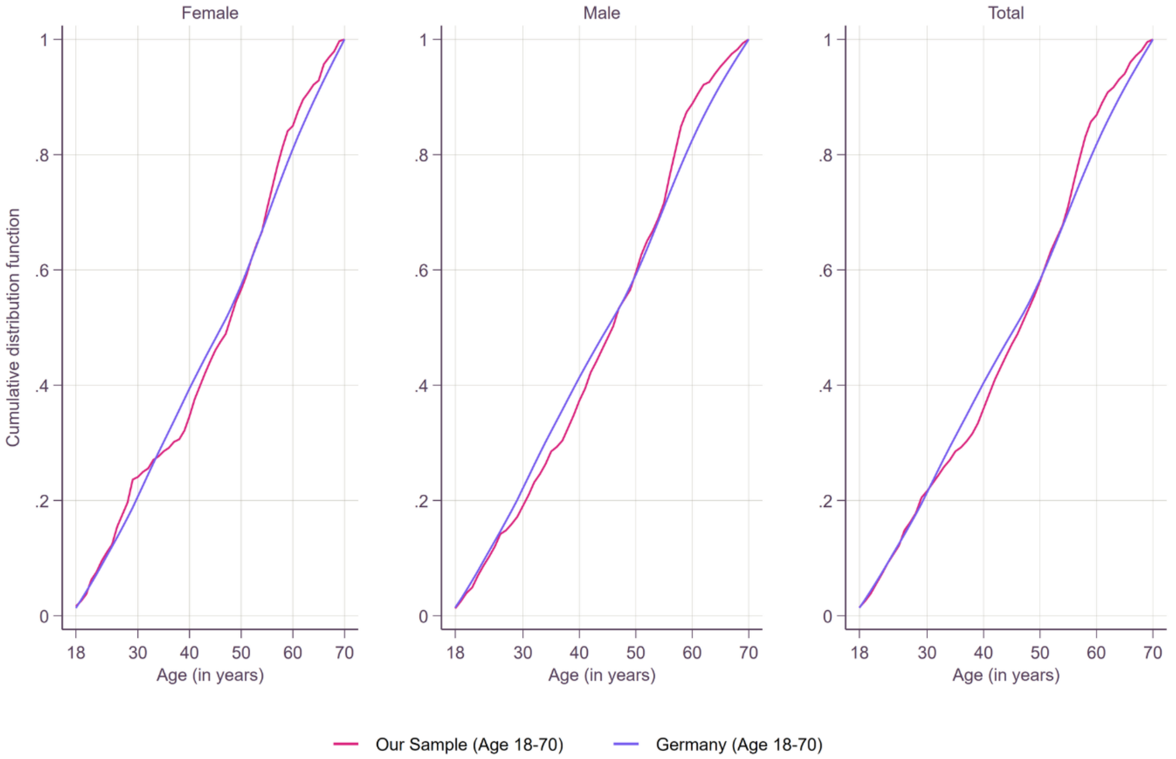

Fig S5. Age distribution: sample vs. census. Comparison of cumulative age distribution in our sample to that of the German population. Where the inclination of our sample-line (blue) is greater than that of the German population-line (orange) age is oversampled.

Table S6. *Sample means vs. census data*

Variable	Study sample			Germany
	N	Mean	SD	Mean
Age (18-70)	1,308	44.98	14.03	45.030
Female (18-70)	1,308	0.51	0.50	0.496
Abitur	1,324	0.54	0.50	0.335
Household Size				
Single	1,324	0.17	0.38	0.406
2 Person	1,324	0.32	0.47	0.34
3 Person	1,324	0.26	0.44	0.121
4 Person	1,324	0.13	0.33	0.098
5 Person or more	1,324	0.11	0.32	0.035

Notes: Summary of age, female, higher education (abitur), and household size of our sample and the German census. As our participants in our sample are mainly 18 to 70 years old we confine ourself to a comparison of our sample with the German population for that age bracket in terms of age and female. As we did not find age specific data fitting our dataset we compare education (Abitur) and Household house for the full age distribution.

Table S7. *Study sample vs. unvaccinated population*

Variable	Study sample		Unvaccinated population June 2, 2021	
	N	Percent	N	Percent
Age (18-59)	1,121	84.66%	27,276,784	82.72%
Age (60+)	203	15.34%	5,699,382	17.28%
Observations	1,324		32,976,166	

Notes: We compare our sample from June 2, 2021, to the unvaccinated population of Germany age 18 and above using publicly available vaccination data provided by the Robert Koch Institute (RKI) (https://raw.githubusercontent.com/robert-koch-institut/COVID-19-Impfungen_in_Deutschland/master/Aktuell_Deutschland_Landkreise_COVID-19-Impfungen.csv") and the 2020 census data provided by the German Statistical Office (https://www-genesis.destatis.de/).
