# Supplementary material for: Repeated information of benefits reduces COVID-19 vaccination hesitancy: Experimental evidence from Germany
Source: PLoS One. 2022 Jun 28;17(6):e0270666. doi: 10.1371/journal.pone.0270666 (PMC9239477; doi:10.1371/journal.pone.0270666)
Supplement: S9 Appendix — (PDF) [file pone.0270666.s009.pdf]

S9 Appendix. Additional results and robustness checks

This section provides additional results and robustness checks of our findings. The structure of this sections follows the outline of the main manuscript starting with the survey experiment, continuing with the findings from the balanced panel, and concluding with a detailed look at vaccination hesitancy.

Survey experiment

Vaccination intentions

Table S8. *Treatment effects vaccination intentions*

		mRNA			Vector	
VARIABLES	(1)	(2)	(3)	(4)	(5)	(6)
T1: Debunk	0.03	0.01	-0.02	0.16	0.11	0.06
	(0.16)	(0.16)	(0.10)	(0.16)	(0.15)	(0.13)
T2: Benefits	0.16	0.09	0.11	0.20	0.13	0.11
	(0.15)	(0.15)	(0.09)	(0.16)	(0.15)	(0.13)
Socio-economics						
Female (=1)		-0.22*	-0.11		-1.10***	-0.97***
		(0.13)	(0.08)		(0.13)	(0.11)
Age: 32-44 (=1)		-0.23	0.21		-0.34	-0.08
		(0.23)	(0.16)		(0.22)	(0.19)
Age: 45-52 (=1)		-0.10	-0.12		0.02	-0.00
		(0.20)	(0.12)		(0.20)	(0.17)
Age: 53-58 (=1)		-0.25	-0.13		0.04	0.10
		(0.19)	(0.12)		(0.19)	(0.16)
Age: 59-81 (=1)		-0.71**	-0.44***		0.06	0.20
		(0.31)	(0.16)		(0.29)	(0.23)
Secondary school: ‘Realschulabschluss’ (=1)		0.42*	0.28*		0.16	0.08
		(0.24)	(0.15)		(0.21)	(0.18)
High school (=1)		0.84***	0.31**		0.66***	0.30
		(0.25)	(0.15)		(0.22)	(0.18)
University degree (=1)		0.81***	0.19		0.85***	0.41**
		(0.25)	(0.16)		(0.23)	(0.19)
Adjusted HH income		0.08**	-0.01		0.08**	0.01
		(0.04)	(0.02)		(0.04)	(0.03)
Married (=1)		-0.09	-0.13		-0.15	-0.16
		(0.14)	(0.09)		(0.13)	(0.11)
Reasons						
Vaccination inaction (=1)			-1.10***			-0.66***
			(0.10)			(0.13)
Denied other vaccine (=1)			-0.53***			-0.27**
			(0.13)			(0.14)
Index: COVID-19 risk perception std.)			0.21***			-0.02
			(0.05)			(0.07)
Index: Emotional response (std.)			0.07			0.03
			(0.05)			(0.07)
Net anticipated regret (std.)			1.35***			1.01***
			(0.05)			(0.07)
Index: Dogmatism (std.)			-0.04			-0.02

			(0.04)			(0.06)
Constant	5.12***	4.58***	5.77***	3.23***	3.12***	3.89***
	(0.09)	(0.29)	(0.18)	(0.09)	(0.28)	(0.25)
Observations	1,324	1,324	1,324	1,324	1,324	1,324
R-squared	0.00	0.03	0.64	0.00	0.09	0.36
Adjusted R-squared	-0.001	0.025	0.640	0.000	0.080	0.349
F-test: Socio-economics		0.000	0.001		0.000	0.000
F-test: Reasons			0.000			0.000

Notes: The dependent variable is the intention to get vaccinated, separately for mRNA and vector vaccines, measured using a 7-point Likert scale. Estimates are obtained from multiple least square regressions with robust standard errors in parentheses: *** p<.01, ** p<.05, * p<.1.

Table S9. *Heterogeneous treatments effects: mRNA*

	Intention mRNA (1-7)					
VARIABLES	(1)	(2)	(3)	(4)	(5)	(6)
T1: Debunk	0.20**	0.07	0.15	0.12	-0.10	0.03
	(0.10)	(0.16)	(0.15)	(0.15)	(0.11)	(0.16)
T2: Benefits	-0.01	0.13	0.23	0.22	0.03	0.10
	(0.10)	(0.16)	(0.14)	(0.14)	(0.10)	(0.15)
Vaccination inaction (=1)	-2.68***					
	(0.15)					
T1 * Vaccination inaction	-0.23					
	(0.25)					
T2 * Vaccination inaction	0.28					
	(0.25)					
Denied other vaccines (=1)		-1.54***				
		(0.27)				
T1 * Denied		-1.06**				
		(0.50)				
T2 * Denied		-0.36				
		(0.47)				
Index: COVID-19 risk perception (std.)			0.89***			
			(0.08)			
T1 * Risk index			0.16			
			(0.14)			
T2 * Risk index			0.02			
			(0.14)			
Index: Emotional response (std.)				0.86***		
				(0.09)		
T1 * Emotional response				0.17		
				(0.15)		
T2 * Emotional response				-0.02		
				(0.15)		
Net anticipated regret (std.)					1.78***	
					(0.06)	
T1 * Net anticipated regret					0.01	
					(0.10)	
T2 * Net anticipated regret					0.05	
					(0.09)	
Index: Dogmatism (std.)						-0.15
						(0.10)
T1 * Dogmatism						-0.13
						(0.17)
T2 * Dogmatism						-0.15
						(0.15)
Constant	6.50***	4.81***	4.51***	4.59***	5.02***	4.61***
	(0.25)	(0.28)	(0.26)	(0.27)	(0.19)	(0.29)
Observations	1,324	1,324	1,324	1,324	1,324	1,324
Adjusted R-squared	0.35	0.10	0.18	0.16	0.58	0.03
Interaction: T1 sig.	0.000	0.000	0.000	0.000	0.000	0.099
Interaction: T2 sig.	0.000	0.000	0.000	0.000	0.000	0.025

Notes: The dependent variable is the intention to get vaccinated with a mRNA vaccine, measured using a 7-point Likert scale. Estimates are obtained from multiple least square regressions with robust standard errors in parentheses: *** p<.01, ** p<.05, * p<.1.

Table S10. *Heterogeneous effects: Vector*

	Intention Vector (1-7)					
VARIABLES	(1)	(2)	(3)	(4)	(5)	(6)
T1: Debunk	0.05	0.12	0.19	0.17	0.04	0.13
	(0.22)	(0.17)	(0.15)	(0.15)	(0.13)	(0.15)
T2: Benefits	-0.07	0.18	0.20	0.19	0.09	0.14
	(0.22)	(0.17)	(0.15)	(0.15)	(0.13)	(0.15)
Vaccination inaction (=1)	-1.88***					
	(0.16)					
T1 * Vaccination inaction	0.20					
	(0.28)					
T2 * Vaccination inaction	0.44					
	(0.28)					
Denied other vaccines (=1)		-0.98***				
		(0.22)				
T1 * Denied		-0.38				
		(0.37)				
T2 * Denied		-0.38				
		(0.35)				
Index: COVID-19 risk perception (std.)			0.44***			
			(0.08)			
T1 * Risk index			0.10			
			(0.15)			
T2 * Risk index			0.07			
			(0.14)			
Index: Emotional response (std.)				0.38***		
				(0.09)		
T1 * Emotional response				0.23		
				(0.16)		
T2 * Emotional response				0.28*		
				(0.15)		
Net anticipated regret (std.)					1.25***	
					(0.07)	
T1 * Net anticipated regret					-0.09	
					(0.12)	
T2 * Net anticipated regret					-0.05	
					(0.12)	
Index: Dogmatism (std.)						-0.15*
						(0.09)
T1 * Dogmatism						-0.02
						(0.16)
T2 * Dogmatism						0.06
						(0.15)
Constant	4.42***	3.27***	3.08***	3.13***	3.41***	3.14***
	(0.27)	(0.27)	(0.26)	(0.26)	(0.23)	(0.27)
Observations	1,324	1,324	1,324	1,324	1,324	1,324
Adjusted R-squared	0.21	0.11	0.12	0.12	0.33	0.08
Interaction: T1 sig.	0.000	0.000	0.000	0.000	0.000	0.194
Interaction: T2 sig.	0.000	0.000	0.000	0.000	0.000	0.266

Notes: The dependent variable is the intention to get vaccinated with a vector vaccine, measured using a 7-point Likert scale. Estimates are obtained from multiple least square regressions with robust standard errors in parentheses: *** p<.01, ** p<.05, * p<.1.

We thank an anonymous reviewer for the suggestion to investigate heterogeneous treatment effects depending on participants' priority group status. In Germany, vaccination priorities were ranked by age, medical condition, and profession (health care, nursing, etc.). At the time of the survey experiment, prioritization was lifted and all people above the age of 16 could apply for a vaccination appointment at vaccination centers or their general practitioner. Officially, vaccination prioritization ended in Germany on June 7th, but because of the high demand, appointments were organized several weeks in advance. In our sample, 33 participants (2.5%) reported to be in the first prioritization group (highest priority), 129 (9.7%) in the second priority group (high priority), and 342 (25,8%) in the third group (increased priority). Thus, about 38% of participants in the survey experiment could potentially be more hesitant than the rest, because they already had the chance to get vaccinated, or at least to get an appointment, due to their priority status.

First, looking at how vaccination intentions vary across priority groups (see Fig S6A), we find that intentions are indeed slightly lower for participants in group 1 (the highest priority group) compared to participants without priority status (T-Test diff. = -.93, t_{851} = -2.15, p = .03). Importantly, participants in group 2 (high priority) have similar intentions (T-Test diff. = .09, t_{947} = .41, p = .68) and participants in group 3 (increased priority) even have significant higher intentions compared to participants without priority status (T-Test diff. = 1.42, $t_{1,160}$ = 9.96, $p < .001$). Second, we analyze whether participants respond differently to the treatments in the survey experiment depending on their priority status (see Fig S6B). Given the low number of observations in each treatment condition of participants in the first two priority groups, we opted for a binary specification of priority status (which equals 1 if a respondent is in priority group 1, 2, or 3 and equals 0 if a respondent has no priority status). We find that among the group of participants with priority status, intentions to get vaccinated are slightly higher in the benefits treatment compared to the control group (ß = .37, p = .07, 95%CI: -.03 to .77). In addition, we find that none of the treatments significantly affected vaccination intentions among participants with priority status. This could be seen as suggestive evidence that the benefits treatment increased vaccination intentions only among

participants without priority status (but only at the 10% significance level). When controlling for the same set of covariates as in Table S8 the effect is not statistically significant when we only include participants without priority status in the regression (see Table S11, model 3).

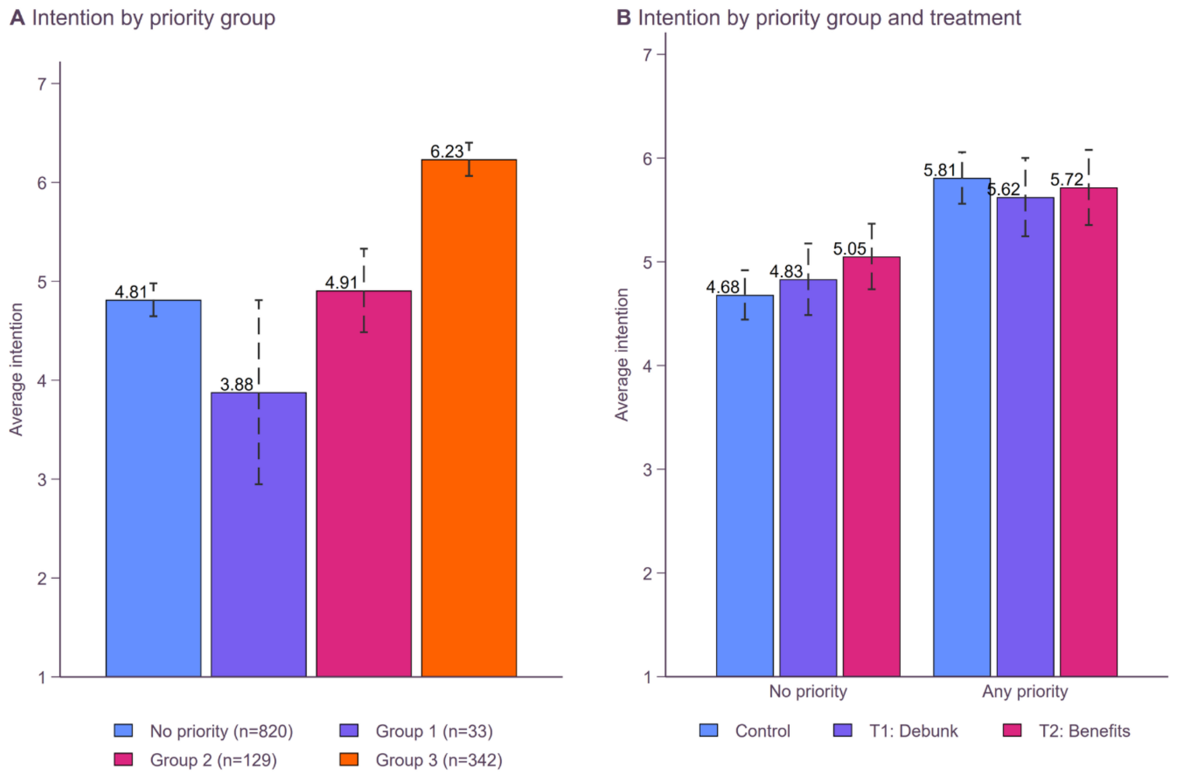

Fig. S6. Vaccination intentions. Panel A shows average intentions to get vaccinated with an mRNA vaccine across priority groups. Panel B shows treatment effects by priority status with a binary specification that equals 1 if the participant reported being in one of the three priority groups and 0 otherwise. Dashed lines indicate 95% confidence intervals.

Taken together, these findings speak against our prior belief that unvaccinated participants with priority status are systematically more hesitant than the rest of the sample. It might be the case that participants with priority group status still faced barriers in getting their vaccination. For example, they might have struggled to make an appointment which often required either computer skills or waiting in long phone queues. Especially older participants, who predominantly made up the highest priority groups, might have struggled with this. In the survey experiment, 70% of participants with priority status reported that they have a vaccination appointment (27%) or are on a waiting list (43%). Thus, it seems that most participants with priority status in our sample only have or had to delay their vaccination but were still getting their vaccination faster than participants without the priority status (only 8% of them had an appointment and 26% were on a waiting list).

Table S11. *Treatment effects vaccination intentions: Only respondents without prioritization status*

VARIABLES	(1)	mRNA (2)	(3)
T1: Debunk	0.15	0.11	0.03
	(0.21)	(0.21)	(0.13)
T2: Benefits	0.37*	0.30	0.19
	(0.20)	(0.20)	(0.12)
Socio-economics			
Female (=1)		-0.23	-0.16
		(0.17)	(0.11)
Age: 32-44 (=1)		-0.49*	0.03
		(0.29)	(0.21)
Age: 45-52 (=1)		-0.35	-0.25
		(0.26)	(0.16)
Age: 53-58 (=1)		-0.52**	-0.29*
		(0.24)	(0.16)
Age: 59-81 (=1)		-1.97***	-0.78***
		(0.42)	(0.27)
Secondary school: 'Realschulabschluss' (=1)		0.48	0.17
		(0.31)	(0.20)
High school (=1)		0.80**	0.18
		(0.32)	(0.21)
University degree (=1)		0.93***	0.07
		(0.32)	(0.21)
Adjusted HH income		0.05	-0.02
		(0.05)	(0.03)
Married (=1)		-0.10	-0.17
		(0.19)	(0.12)
Reasons			
Vaccination inaction (=1)			-0.92***
			(0.12)
Denied other vaccine (=1)			-0.55***
			(0.18)
Index: COVID-19 risk perception std.)			0.23***
			(0.07)
Index: Emotional response (std.)			0.08
			(0.07)
Net anticipated regret (std.)			1.40***
			(0.06)
Index: Dogmatism (std.)			-0.03
			(0.05)
Constant	4.68***	4.48***	5.90***
	(0.12)	(0.37)	(0.25)
Observations	820	820	820
R-squared	0.00	0.06	0.64
Adjusted R-squared	0.002	0.048	0.628
F-test: Socio-economics		0.000	0.007
F-test: Reasons			0.000

Notes: The dependent variable is the intention to get vaccinated, separately for mRNA and vector vaccines, measured using a 7-point Likert scale. Estimates are obtained from multiple least square regressions with robust standard errors in parentheses: *** p<.01, ** p<.05, * p<.1.

Psychological antecedents of vaccination (5C)

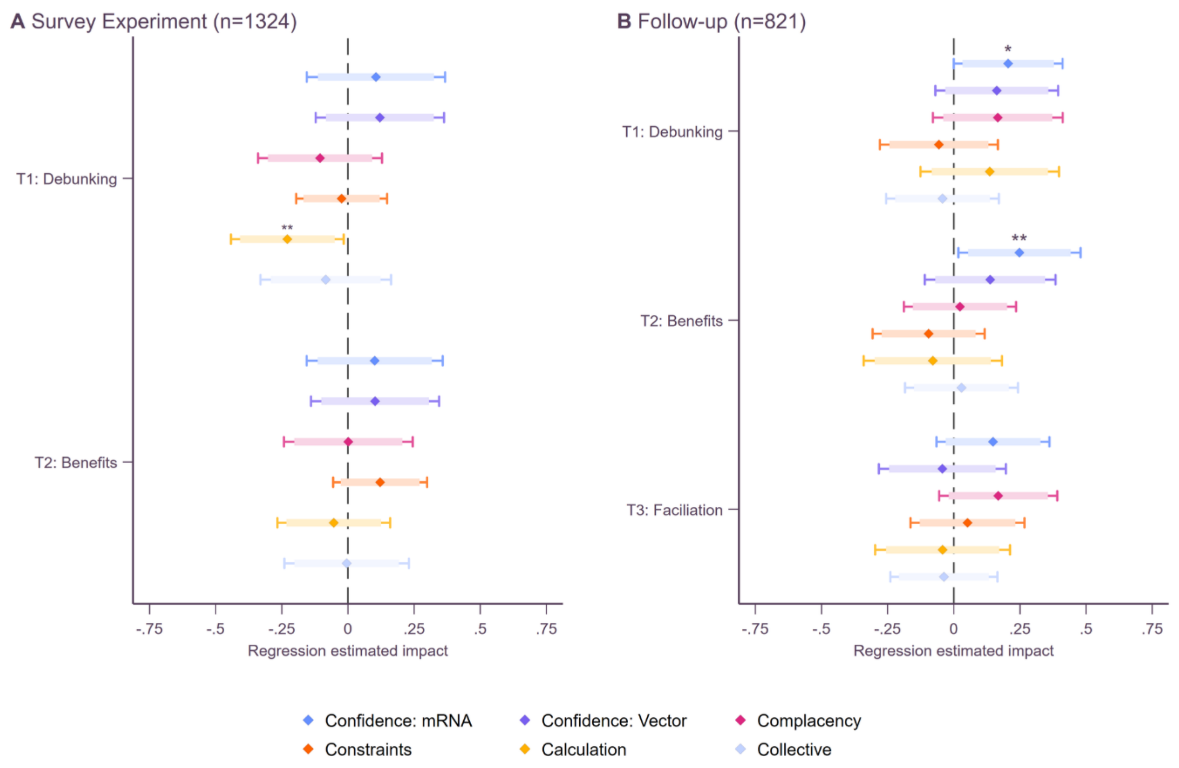

Fig S7. Treatment effects on 5c. Panel A shows regression estimates with each 5C factor as the dependent variable in the survey experiment. Panel B shows the results on each 5C factor in the follow-up survey. Estimates are obtained from linear probability models controlling for gender, age, education, adjusted household income, and marital status. Heteroskedasticity robust standard errors were used to compute 95% (thin bars) and 90% (thick bars) confidence intervals.

Table S12. *Treatment effects on 5C: Wave 1*

VARIABLES	Confidence: mRNA<br>(1)	Confidence: Vector<br>(2)	Complacency<br>(3)	Constraints<br>(4)	Calculation<br>(5)	Collective<br>(6)
T1: Debunking (=1)	0.11	0.12	-0.11	-0.02	-0.23**	-0.08
	(0.13)	(0.12)	(0.12)	(0.09)	(0.11)	(0.13)
T2: Benefits (=1)	0.10	0.10	0.00	0.12	-0.05	-0.00
	(0.13)	(0.12)	(0.12)	(0.09)	(0.11)	(0.12)
Socio-economics:						
Female (=1)	-0.30***	-0.53***	-0.13	-0.22***	0.17*	-0.01
	(0.11)	(0.10)	(0.10)	(0.07)	(0.09)	(0.10)
Age: 32-44 (=1)	-0.54***	-0.39**	0.22	-0.35**	0.28*	-0.21
	(0.19)	(0.18)	(0.18)	(0.14)	(0.16)	(0.18)
Age: 45-52 (=1)	-0.43***	-0.04	-0.25	-0.35***	0.40***	0.11
	(0.17)	(0.16)	(0.16)	(0.13)	(0.14)	(0.16)
Age: 53-58 (=1)	-0.54***	-0.16	-0.50***	-0.77***	0.55***	0.13
	(0.16)	(0.15)	(0.14)	(0.11)	(0.13)	(0.14)
Age: 59-81 (=1)	-0.76***	-0.40*	-0.17	-0.99***	0.68***	-0.09
	(0.25)	(0.24)	(0.23)	(0.14)	(0.19)	(0.24)
Secondary school (=1)	0.24	0.20	0.11	-0.22*	0.12	0.01
	(0.20)	(0.18)	(0.17)	(0.13)	(0.16)	(0.18)
High school (=1)	0.64***	0.71***	-0.27	-0.31**	0.22	0.44**
	(0.21)	(0.19)	(0.18)	(0.13)	(0.16)	(0.19)
University degree (=1)	0.73***	0.91***	-0.14	-0.20	0.26	0.47**
	(0.21)	(0.19)	(0.18)	(0.14)	(0.17)	(0.19)
Adjusted HH income	0.06**	0.04	-0.03	-0.03	-0.04*	0.06**
	(0.03)	(0.03)	(0.03)	(0.02)	(0.03)	(0.03)
Married (=1)	-0.00	-0.07	0.15	0.02	0.08	-0.03
	(0.12)	(0.11)	(0.11)	(0.08)	(0.09)	(0.11)
Constant	4.53***	3.64***	3.63***	3.06***	4.66***	4.56***
	(0.24)	(0.23)	(0.21)	(0.17)	(0.20)	(0.22)
Observations	1,324	1,324	1,324	1,324	1,324	1,324
Adjusted R-squared	0.04	0.06	0.02	0.06	0.02	0.01
F-Test: Socio-economics	0.000	0.000	0.000	0.000	0.001	0.001

Notes: The dependent variable are the 5C factors, measured using several 7-point Likert items in the survey experiment. Estimates are obtained from multiple least square regressions with robust standard errors in parentheses: *** p<.01, ** p<.05, * p<.1.

Table S13. *Treatment effects on 5C: Wave 2*

VARIABLES	Confidence: mRNA (1)	Confidence: Vector (2)	Complacency (3)	Constraints (4)	Calculation (5)	Collective (6)
T1: Debunking (=1)	0.21*	0.16	0.17	-0.06	0.14	-0.04
	(0.10)	(0.12)	(0.12)	(0.11)	(0.13)	(0.11)
T2: Benefits (=1)	0.25**	0.14	0.02	-0.10	-0.08	0.03
	(0.12)	(0.13)	(0.11)	(0.11)	(0.13)	(0.11)
T3: Facilitation (=1)	0.15	-0.04	0.17	0.05	-0.04	-0.04
	(0.11)	(0.12)	(0.11)	(0.11)	(0.13)	(0.10)
Baseline: Outcome	0.87***	0.84***	0.78***	0.46***	0.55***	0.80***
	(0.02)	(0.02)	(0.02)	(0.05)	(0.04)	(0.02)
Socio-economics:						
Female (=1)	-0.01	-0.02	-0.03	-0.18**	0.21**	0.12
	(0.08)	(0.09)	(0.08)	(0.08)	(0.10)	(0.08)
Age: 32-44 (=1)	0.12	-0.18	-0.18	-0.17	0.21	0.09
	(0.16)	(0.18)	(0.21)	(0.20)	(0.19)	(0.17)
Age: 45-52 (=1)	0.10	-0.06	-0.25	-0.41***	0.27	0.24*
	(0.14)	(0.17)	(0.16)	(0.16)	(0.17)	(0.14)
Age: 53-58 (=1)	0.15	-0.01	-0.21	-0.38***	0.44***	0.24*
	(0.13)	(0.16)	(0.16)	(0.14)	(0.15)	(0.13)
Age: 59-81 (=1)	0.16	0.12	-0.15	-0.16	0.25	0.35**
	(0.17)	(0.20)	(0.21)	(0.20)	(0.22)	(0.16)
Secondary school (=1)	-0.02	-0.20	-0.26*	0.14	0.22	0.09
	(0.13)	(0.15)	(0.15)	(0.13)	(0.18)	(0.13)
High school (=1)	0.14	-0.12	-0.24	0.18	0.43**	0.15
	(0.14)	(0.17)	(0.17)	(0.14)	(0.19)	(0.14)
University degree (=1)	0.19	-0.17	-0.35**	0.14	0.42**	0.20
	(0.14)	(0.17)	(0.16)	(0.14)	(0.19)	(0.14)
Adjusted HH income	0.01	-0.02	-0.00	-0.00	0.04*	-0.04**
	(0.02)	(0.02)	(0.02)	(0.02)	(0.03)	(0.02)
Married (=1)	0.11	0.06	-0.01	-0.01	0.06	-0.05
	(0.08)	(0.09)	(0.09)	(0.09)	(0.10)	(0.08)
Constant	0.19	0.99***	1.07***	1.09***	1.40***	1.01***
	(0.21)	(0.24)	(0.26)	(0.22)	(0.29)	(0.21)
Observations	821	821	821	821	821	821
Adjusted R-squared	0.72	0.63	0.60	0.23	0.32	0.66
F-Test: Socio-economics	0.459	0.591	0.740	0.037	0.005	0.325

Notes: The dependent variable are the 5C factors, measured using several 7-point Likert items in the follow-up survey. Estimates are obtained from multiple least square regressions with robust standard errors in parentheses: *** p<.01, ** p<.05, * p<.1.

Main result without exclusion of participants

As a relatively high share of participants had to be excluded from the analysis due to data quality issues and as those excluded do differ from those not excluded (see S4 Appendix), we decided to run the main regressions again without exclusions. Leaving all observations in the sample does not affect the results obtained for the regression of intention to get vaccinated on treatments as shown in Table S13. We report only the main results here, i.e. the effect of treatments on the vaccination intention. Further analysis testing the effects of heterogeneous effects and the effect of the treatments on the 5Cs have been carried out for the sample without exclusions as well. We also tested if excluded participants reacted differently to the treatments by interacting treatment dummies with the exclusion dummy. For all these tests, the results do not change coefficients sign or statistical significance. Results are not reported here but can be received upon request.

Table S14. *Treatment effects vaccination intentions (without exclusion)*

		mRNA			Vector	
VARIABLES	(1)	(2)	(3)	(4)	(5)	(6)
	-0.01	-0.01	-0.05	0.11	0.07	0.01
T1: Debunk	(0.15)	(0.15)	(0.09)	(0.14)	(0.14)	(0.12)
	0.18	0.11	0.13	0.10	0.04	0.03
T2: Benefits	(0.14)	(0.14)	(0.09)	(0.15)	(0.14)	(0.12)
Socio-economics						
Female (=1)		-0.12	-0.02		-1.08***	-0.97***
		(0.12)	(0.08)		(0.11)	(0.10)
Age: 32-44 (=1)		-0.07	0.25*		-0.28	-0.10
		(0.20)	(0.14)		(0.19)	(0.16)
Age: 45-52 (=1)		0.10	0.02		-0.11	-0.18
		(0.18)	(0.12)		(0.18)	(0.15)
Age: 53-58 (=1)		-0.13	-0.04		-0.12	-0.09
		(0.18)	(0.11)		(0.17)	(0.14)
Age: 59-81 (=1)		-0.58**	-0.40**		-0.12	-0.06
		(0.28)	(0.16)		(0.26)	(0.21)
Secondary school: 'Realschulabschluss' (=1)		0.35	0.22		0.06	-0.01
		(0.22)	(0.13)		(0.19)	(0.16)
High school (=1)		0.78***	0.27*		0.57***	0.23
		(0.22)	(0.14)		(0.20)	(0.17)
University degree (=1)		0.67***	0.11		0.70***	0.32*
		(0.23)	(0.14)		(0.21)	(0.18)
Adjusted HH income		0.10***	0.01		0.08**	0.02
		(0.03)	(0.02)		(0.04)	(0.03)
Married (=1)		-0.10	-0.17**		-0.01	-0.06
		(0.13)	(0.08)		(0.12)	(0.11)
Reasons						
Vaccination inaction (=1)			-1.06***			-0.69***
			(0.09)			(0.12)
Denied other vaccine (=1)			-0.47***			-0.19
			(0.12)			(0.13)
Index: COVID-19 risk perception std.)			0.19***			-0.05
			(0.05)			(0.06)
Index: Emotional response (std.)			0.10**			0.08
			(0.05)			(0.07)

Net anticipated regret (std.)			1.31***			0.96***
			(0.05)			(0.06)
Index: Dogmatism (std.)			-0.02			0.05
			(0.04)			(0.05)
Constant	5.04***	4.35***	5.55***	3.29***	3.28***	4.08***
	(0.08)	(0.26)	(0.17)	(0.08)	(0.24)	(0.22)
Observations	1,532	1,531	1,531	1,532	1,531	1,531
R-squared	0.00	0.03	0.62	0.00	0.09	0.35
Adjusted R-squared	0.00	0.02	0.61	0.00	0.08	0.34
F-test: Socio-economics		0.00	0.00		0.00	0.00
F-test: Reasons			0.00			0.00

Notes: The dependent variable is the intention to get vaccinated, separately for mRNA and vector vaccines, measured using a 7-point Likert scale. Estimates are obtained from multiple least square regressions with robust standard errors in parentheses: *** p<.01, ** p<.05, * p<.1.

Vaccination (in)action

Table S15. *Treatment effects on inaction: Balanced Panel*

	Wave 2: Vaccination inaction (=1)					
VARIABLES	(1)	(2)	(3)	(4)	(5)	(6)
T1: Debunk (=1)	-0.03	-0.03	-0.01	0.00	-0.00	0.03
	(0.04)	(0.04)	(0.03)	(0.02)	(0.02)	(0.03)
T2: Benefits (=1)	-0.08**	-0.08**	-0.06*	0.01	0.03	0.04
	(0.04)	(0.04)	(0.03)	(0.02)	(0.02)	(0.03)
T3: Facilitator (=1)	-0.05	-0.05	-0.04	-0.01	-0.01	-0.01
	(0.04)	(0.04)	(0.03)	(0.02)	(0.02)	(0.03)
Baseline: Vaccination inaction (=1)	0.49***	0.48***	0.26***	0.56***	0.56***	0.34***
	(0.02)	(0.02)	(0.03)	(0.05)	(0.05)	(0.05)
Interactions:						
T1 * Baseline				-0.05	-0.05	-0.08
				(0.07)	(0.07)	(0.06)
T2 * Baseline				-0.17**	-0.19***	-0.19***
				(0.07)	(0.07)	(0.07)
T3 * Baseline				-0.07	-0.08	-0.05
				(0.07)	(0.07)	(0.06)
Constant	0.06**	0.09	0.18***	0.02	0.05	0.14**
	(0.02)	(0.07)	(0.06)	(0.01)	(0.06)	(0.06)
Socio-economics	N	Y	Y	N	Y	Y
Other reasons	N	N	Y	N	N	Y
Observations	821	821	821	821	821	821
R-squared	0.30	0.31	0.44	0.30	0.32	0.45

Notes: Linear probability model with robust standard errors in parentheses: *** p<.01, ** p<.05, * p<.1

Table S16. *Robustness check using non-linear Probit models and computing margins*

	Wave 2: Vaccination inaction (=1)					
VARIABLES	(1)	(2)	(3)	(4)	(5)	(6)
T1: Debunk (=1)	-0.02	-0.03	-0.02			
	(0.04)	(0.04)	(0.03)			
T2: Benefits (=1)	-0.07**	-0.08**	-0.07**			
	(0.04)	(0.04)	(0.03)			
T3: Facilitator (=1)	-0.05	-0.05	-0.03			
	(0.04)	(0.04)	(0.03)			
Baseline: Vaccination inaction (=1)	0.49***	0.49***	0.27***			
	(0.02)	(0.02)	(0.03)			
Interactions:						
T1 * Baseline				-0.05	-0.06	-0.07
				(0.07)	(0.07)	(0.08)
T2 * Baseline				-0.17**	-0.19***	-0.17**
				(0.07)	(0.07)	(0.07)
T3 * Baseline				-0.07	-0.08	-0.04
				(0.07)	(0.07)	(0.07)
	N	Y	Y	N	Y	Y
Socio-economics	N	N	Y	N	N	Y
Other reasons	0.30	0.31	0.45	0.30	0.32	0.45
Pseudo R-squared	821	821	821	821	821	821
Observations	-0.02	-0.03	-0.02			

Notes: Non-linear probability model using Probit link function and computing average marginal effects with robust standard errors in parentheses: *** p<.01, ** p<.05, * p<.1

Table S17. *Robustness check linear model (without exclusion)*

	Wave 2: Vaccination inaction (=1)					
VARIABLES	(1)	(2)	(3)	(4)	(5)	(6)
T1: Debunk (=1)	-0.01	-0.01	-0.01	0.01	0.01	0.01
	(0.03)	(0.03)	(0.03)	(0.02)	(0.02)	(0.02)
T2: Benefits (=1)	-0.08**	-0.08**	-0.08**	0.02	0.03	0.03
	(0.03)	(0.03)	(0.03)	(0.02)	(0.02)	(0.02)
T3: Facilitator (=1)	-0.05	-0.05	-0.05	0.01	0.00	0.00
	(0.03)	(0.03)	(0.03)	(0.02)	(0.02)	(0.02)
Baseline: Vaccination inaction (=1)						
	0.48***	0.47***	0.47***	0.56***	0.56***	0.56***
Interactions:	(0.02)	(0.02)	(0.02)	(0.04)	(0.04)	(0.04)
T1 * Baseline				-0.03	-0.03	-0.03
				(0.06)	(0.06)	(0.06)
T2 * Baseline				-0.19***	-0.21***	-0.21***
				(0.06)	(0.06)	(0.06)
T3 * Baseline				-0.10	-0.11*	-0.11*
				(0.06)	(0.06)	(0.06)
Constant	0.06***	0.07	0.07	0.02	0.03	0.03
	-0.01	-0.01	-0.01	0.01	0.01	0.01
Socio-economics	N	Y	Y	N	Y	Y
Other reasons	N	N	Y	N	N	Y
Observations	987	986	986	987	986	986
R-squared	0.29	0.30	0.30	0.29	0.31	0.31

Notes: Linear probability model with robust standard errors in parentheses: *** p<.01, ** p<.05, * p<.1

Table S18. *Heterogeneous treatment effects as pre-registered*

VARIABLES	(1)	(2)	(3)	(4)	(5)	(6)
T1: Debunk	-0.00	-0.05	-0.06	-0.04	0.00	-0.05
	(0.02)	(0.05)	(0.04)	(0.04)	(0.04)	(0.04)
T2: Benefits	0.03	-0.09**	-0.10**	-0.09**	-0.05	-0.09**
	(0.02)	(0.04)	(0.04)	(0.04)	(0.04)	(0.04)
T3: Facilitation	-0.01	-0.04	-0.04	-0.02	-0.02	-0.04
	(0.02)	(0.05)	(0.04)	(0.04)	(0.04)	(0.05)
Interactions with baseline values						
Vaccination inaction (=1)	0.57***					
	(0.05)					
T1 * Vaccination inaction	-0.05					
	(0.07)					
T2 * Vaccination inaction	-0.20***					
	(0.07)					
T3 * Vaccination inaction	-0.07					
	(0.07)					
Denied other vaccines (=1)		0.18*				
		(0.10)				
T1 * Denied		0.19				
		(0.14)				
T2 * Denied		0.10				
		(0.14)				
T3 * Denied		0.12				
		(0.14)				
Index: COVID-19 risk perception (std.)			-0.14***			
			(0.03)			
T1 * Risk index			-0.01			
			(0.04)			
T2 * Risk index			0.03			
			(0.04)			
T3 * Risk index			-0.00			
			(0.04)			
Index: Emotional response (std.)				-0.13***		
				(0.03)		
T1 * Emotional response				0.00		
				(0.04)		
T2 * Emotional response				0.01		
				(0.04)		
T3 * Emotional response				-0.03		
				(0.04)		
Net anticipated regret (std.)					-0.28***	
					(0.02)	
T1 * Net anticipated regret					0.01	
					(0.03)	
T2 * Net anticipated regret					0.03	
					(0.03)	
T3 * Net anticipated regret					-0.01	
					(0.03)	
Index: Dogmatism (std.)						0.09***
						(0.03)
T1 * Dogmatism						-0.00
						(0.04)
T2 * Dogmatism						-0.06
						(0.04)
T3 * Dogmatism						-0.08
						(0.05)
Constant	0.07	0.46***	0.48***	0.45***	0.39***	0.48***
	(0.06)	(0.08)	(0.08)	(0.08)	(0.07)	(0.08)
Controls: PAP	Y	Y	Y	Y	Y	Y
Observations	821	821	821	821	821	821
Adjusted R-squared	0.30	0.05	0.10	0.10	0.37	0.03
Interaction: T1 sig.	0.00	0.00	0.00	0.00	0.00	0.00
Interaction: T2 sig.	0.00	0.00	0.00	0.00	0.00	0.01
Interaction: T3 sig.	0.00	0.00	0.00	0.00	0.00	0.05

Notes The table shows coefficient estimates from linear probability models of the outcome variable (inaction) on each treatment condition including interactions with the pre-registered measures with heteroskedasticity robust standard errors in parentheses (*** p<.01, ** p<.05, * p<.1). Additional controls as specified in the PAP are gender, age, education, adjusted household income and marital status.

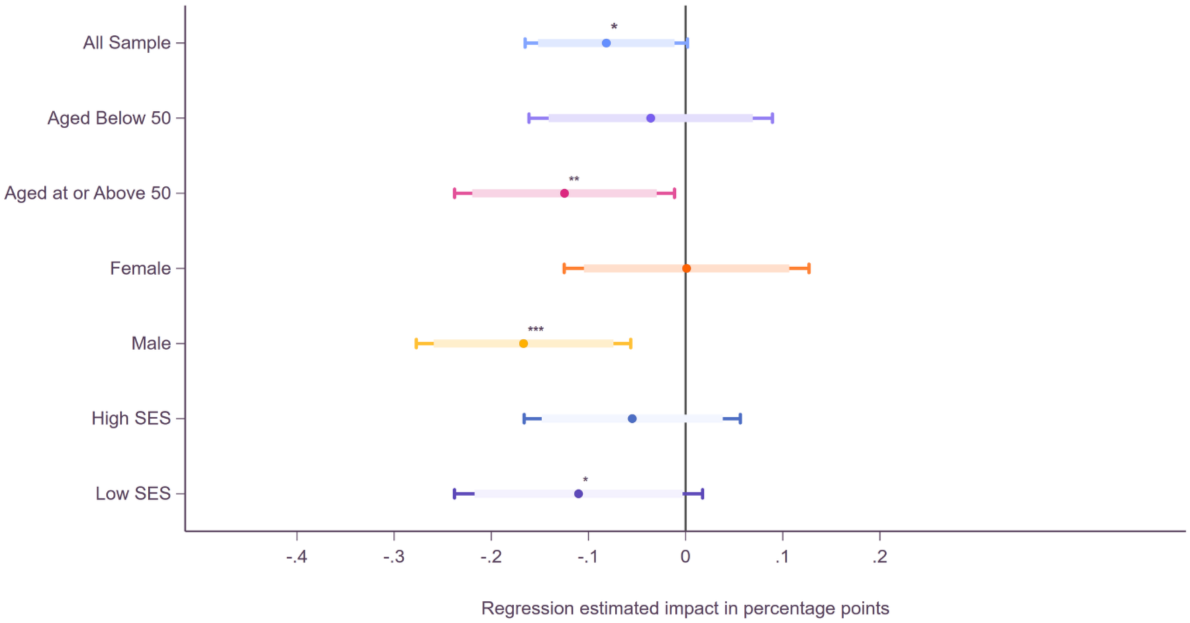

Fig S8. Benefits treatment effects on inaction across socioeconomic groups. The figure shows treatment effects of highlighting benefits across sociodemographic groups, for the entire sample, and separately based on sociodemographic factors. Estimates are always obtained from linear probability models controlling for gender, age, education, adjusted household income, and marital status. Heteroskedasticity robust standard errors were used to compute 95% (thin bars) and 90% (thick bars) confidence intervals.

Table S19. *Determinants of inaction in wave 2*

	Vaccination inaction **(=1)**			
VARIABLES	(1)	(2)	(3)	(4)
T1: Debunking (=1)	-0.05	-0.05	-0.06	-0.06
	(0.07)	(0.07)	(0.06)	(0.06)
T2: Benefits (=1)	-0.16**	-0.18**	-0.13**	-0.13**
	(0.07)	(0.07)	(0.06)	(0.06)
T3: Facilitator (=1)	-0.08	-0.09	-0.08	-0.08
	(0.07)	(0.07)	(0.06)	(0.06)
Socio-economics:				
Female (=1)		0.12**	0.03	0.03
		(0.05)	(0.04)	(0.04)
Age: 30-39 (=1)		-0.05	-0.21**	-0.22**
		(0.10)	(0.09)	(0.10)
Age: 40-49 (=1)		-0.07	-0.16**	-0.16**
		(0.09)	(0.08)	(0.08)
Age: 50-64 (=1)		-0.03	-0.14*	-0.15*
		(0.08)	(0.08)	(0.08)
Age: 65+ (=1)		0.08	-0.13	-0.14
		(0.10)	(0.09)	(0.10)
Secondary school (=1)		-0.04	-0.02	-0.01
		(0.08)	(0.06)	(0.06)
High school (=1)		-0.10	-0.01	0.01
		(0.08)	(0.07)	(0.07)
University degree (=1)		-0.15*	-0.06	-0.05
		(0.09)	(0.07)	(0.07)
Adjusted HH income		-0.02	-0.01	-0.01

		(0.01)	(0.01)	(0.01)
Married (=1)		0.03	0.00	-0.00
		(0.05)	(0.04)	(0.04)
Baseline: Five C				
Confidence: MRNA			-0.10***	-0.10***
			(0.02)	(0.02)
Confidence: VECTOR			0.01	0.01
			(0.03)	(0.03)
Complacency			-0.03*	-0.03*
			(0.01)	(0.02)
Constraints			0.01	0.01
			(0.02)	(0.02)
Calculation			0.04***	0.04**
			(0.02)	(0.02)
Collective			-0.07***	-0.07***
			(0.02)	(0.02)
Reasons				
Denied other vaccine (=1)				-0.06
				(0.06)
Index: COVID-19 risk perception std.)				0.00
				(0.03)
Index: Emotional response (std.)				0.00
				(0.03)
Net anticipated regret (std.)				-0.02
				(0.04)
Index: Dogmatism (std.)				0.03
				(0.02)
Constant	0.58***	0.68***	1.23***	1.25***
	(0.05)	(0.12)	(0.16)	(0.17)
Observations	441	441	441	441
R-squared	0.01	0.06	0.38	0.38
Adjusted R-squared	0.007	0.027	0.352	0.348
F-Test: Treatments	0.108	0.073	0.173	0.205
F-Test: Socio-economics		0.030	0.352	0.381
F-Test: 5C			0.000	0.000
F-Test: Reasons				0.760

Notes: The table shows estimates from linear probability models with inaction as the outcome variable for the subsample of participants that had not taken any actions in the survey experiment. Heteroskedasticity robust standard errors in parentheses (*** p<.01, ** p<.05, * p<.1).
