# Supplementary material for: Repeated information of benefits reduces COVID-19 vaccination hesitancy: Experimental evidence from Germany
Source: PLoS One. 2022 Jun 28;17(6):e0270666. doi: 10.1371/journal.pone.0270666 (PMC9239477; doi:10.1371/journal.pone.0270666)
Supplement: S10 Appendix — (PDF) [file pone.0270666.s010.pdf]

# S10 Appendix. Demand Effects

## Reporting correct batch numbers across treatments

In the follow-up survey, we asked participants who stated that they have been vaccinated once or twice to type in the batch number recorded in their vaccination pass/certificate. Since the survey took place during the time of vaccinations, we assumed participants to have their vaccination pass/certificate in reach. However, not all stated their batch correctly. As no list of batch numbers was available to us, we classified batches as correct or incorrect by the appearance. The logic behind this is that if the higher vaccination rate found in the benefits treatment was indeed due to demand effects, this should also reflect in the batches recorded – i.e., a higher share in the benefits treatment should report correct batch numbers.

On average, of the 557 participants vaccinated once or twice 69% percent reported a correct batch. We do not find a difference of batches reported correctly between treatments (see Fig. S9).

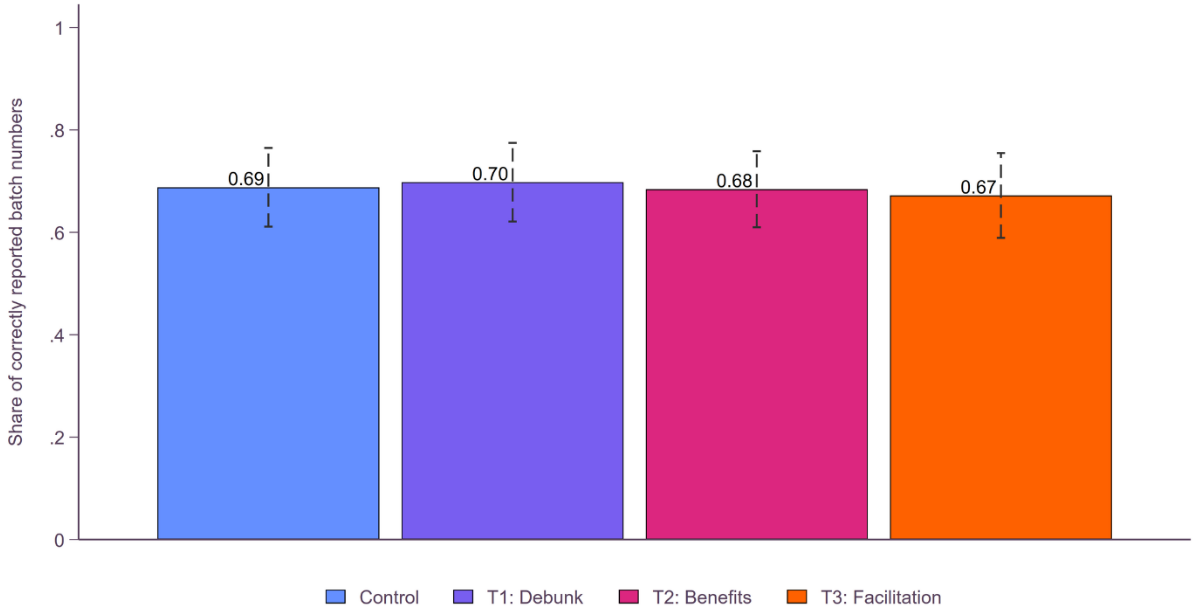


**Fig S9. Share of batch numbers correctly reported by treatment.** Means by treatment group and 95% confidence intervals.
