# Supplementary material for: Repeated information of benefits reduces COVID-19 vaccination hesitancy: Experimental evidence from Germany
Source: PLoS One. 2022 Jun 28;17(6):e0270666. doi: 10.1371/journal.pone.0270666 (PMC9239477; doi:10.1371/journal.pone.0270666)
Supplement: S11 Appendix — (PDF) [file pone.0270666.s011.pdf]

S11 Appendix. External validity

Throughout all treatments, study participants were more likely to get vaccinated within the study period of May 2 and September 18. While this does not affect the internal validity of our results it does make it more difficult to extrapolate our results to the general public.

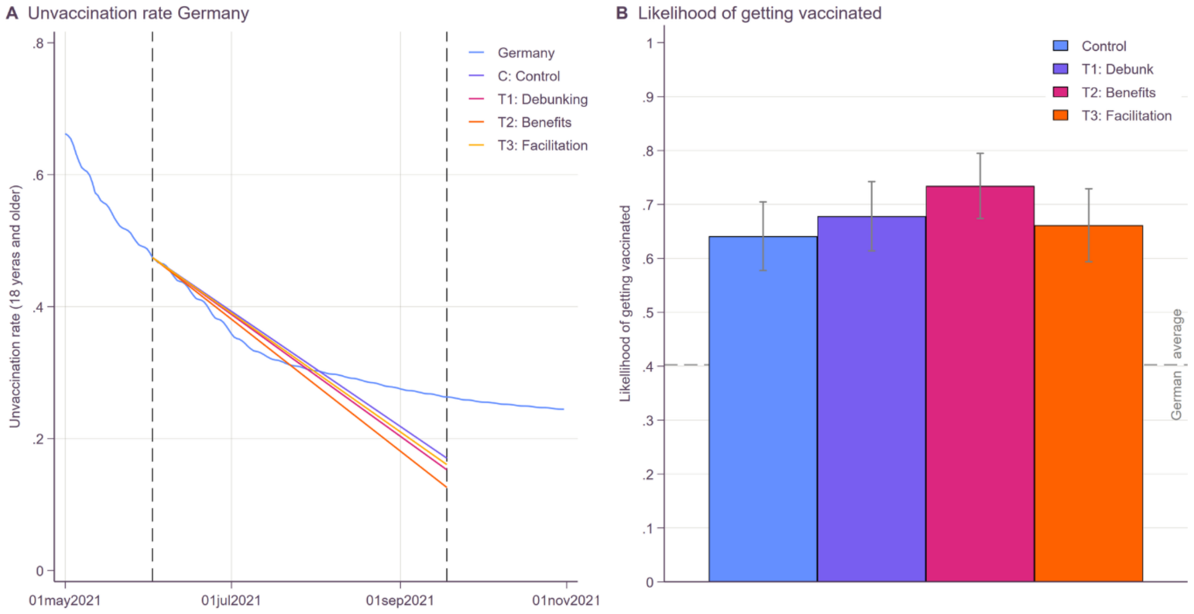

Fig S10. Likelihood of getting vaccinated between May 2 and September 18. Fehler! Verweisquelle konnte nicht gefunden werden. panel A, shows the unvaccinated rate of Germans 18 years and older compared to rates in our study sample for each treatment group. Panel B compares the likelihood of getting vaccinated between May 2 and September 18 for each treatment group. The average likelihood for Germans 18 years and older is indicated by the gray dotted line.
