# Supplementary material for: Repeated information of benefits reduces COVID-19 vaccination hesitancy: Experimental evidence from Germany
Source: PLoS One. 2022 Jun 28;17(6):e0270666. doi: 10.1371/journal.pone.0270666 (PMC9239477; doi:10.1371/journal.pone.0270666)
Supplement: S12 Appendix — (PDF) [file pone.0270666.s012.pdf]

S12 Appendix. Experimental materials

In the following, we present the instructions from the survey as well as the emails sent to the three treatment conditions. The survey and emails were sent in German. An English translation of the text is provided below.

Welcome page and consent form

The text was used for both surveys with the exception of the confirmation of not being vaccinated which was only included in the first survey in May 2021. The welcome page and consent form read as follows:

> Dear Participants,
>
> this survey is part of a research study conducted by Matthias Mayer and colleagues from the University of Marburg. With the survey, we are trying to assess the attitude of the German population towards the topic of corona vaccination.
>
> You should know the following points about the study procedure:
>
> - The survey will take approximately 15 minutes to complete.
> - If you participate in this study, we will invite you to up to two further surveys.
> - Between these surveys, we will send you up to four emails via Respondi AG. Your email address will not be shared with us.
>
> The guidelines of good ethical research require that participants in empirical studies explicitly and comprehensibly agree to participate.
>
> **Voluntariness:** Your participation in this study is voluntary. You are free to discontinue your participation at any time during this study.
>
> **Benefits**: You may not derive any personal benefit from your participation in this study, but the knowledge you obtain may be of value to humanity.
>
> **Compensation**: You will be compensated by Respondi AG for your participation in this survey.
>
> **Anonymity**: Your data is of course confidential, will only be evaluated in anonymous form and will not be passed on to third parties. Demographic information such as age or gender does not allow any clear conclusion to be drawn about your person.
>
> **Questions:** If you have any further questions about this study, please feel free to contact the study director Max Burger at any time (maximilian.burger@wiwi.uni-marburg.de).
>
> I hereby confirm that I ...
>
> - am 18 years of age or older (y/n)
> - have not received any Corona vaccination so far (neither fully nor partially vaccinated) (y/n)
> - agree to receive up to four emails as part of this research project. (y/n)
> - have read and understand the above information. (y/n)
> - wish to participate in this research and proceed with the survey. (y/n)

Survey items

For full transparency and to enable replication we provide all survey items that were used for the main variables of interest. Items have been translated from German to English. All survey items are listed in the order they were recorded in the survey. All items are identical in both surveys in May and September 2021, except for the treatment material which was only included in the first survey in May.

Awareness: Fear of getting infected / consequences of an infection

1. **How likely do you think it is that you will be infected with Corona?**
   Please indicate how likely you think it is that you and others will become infected with Corona.
   [Slider: Low 0% |---| 100% High]
   How likely do you think it is that...
   ... you will become infected with Corona?
   ... one of your friends or family members will become infected with Corona?
   ... the average German becomes infected with Corona?

2. **How severe do you think the effects of Corona would be in your case if you were infected?**
   Please indicate how severe you think the consequences of being infected with Corona would be.
   [Single Choice]
   [ ] No symptoms
   [ ] Mild symptoms (e.g. symptoms similar to a severe cold or flu)
   [ ] Severe symptoms (e.g. acute respiratory distress requiring medical treatment)

3. **How would you rate the likelihood that...** *[Slider: Low 0% |---| 100% High]*
   ...you would suffer long-term consequences after recovering from Corona?
   ...you would die from Corona if you became infected?

4. **Self-evaluation**
   Based on your previous answers, we have summarized your risk assessment in a number. A high number (100) means "I rate Corona as a very high risk to me / society". A low number (0) "I rate Corona as no risk to me / others". The sliders on the scales rest on the values calculated for you.

   If you wish, you can change the values we have calculated according to your assessment. If you agree with the estimation, you can leave it where it rests.

   Own risk: <VALUE1>
   [Slider: No risk to me 0% |---| 100% high risk to me]

   Risk to others: <VALUE2>
   [Slider: No risk to others 0% |---| 100% high risk to others]

5. **What emotions does the thought of the Corona pandemic evoke in you?**
In the following, we would like to know from you what emotions are triggered in you when you think about the Corona pandemic. Please go through the emotions listed below one by one and indicate how strongly you feel each emotion when you think about the Corona Pandemic.
[Not at all 1 |---| 7 Extremly]

Upset
Alarmed
Nervous
Attentive
Anxious

Vaccination Action

6. **Have you already been infected with Corona?**
If yes, please indicate when you became symptom-free. If you are not sure, please provide an approximate date. *[Single Choice]*
Yes
No

7. **What is your current vaccination status?** *[Single Choice]*
[ ] I am fully vaccinated (received all necessary vaccinations).
[ ] Am partially vaccinated (received first of two vaccinations)
[ ] Have not been vaccinated yet, but have a vaccination appointment
[ ] Do not have a vaccination appointment yet, but have registered for a waiting list.
[ ] Have neither a vaccination appointment, nor have I been put on a waiting list

Treatment (only in the first survey in May/June 2021)

Particpants were randomly assigned to one of the conditions: Benefits treatment (T1), Debunk treatment (T2), or Control (C). The two treatment conditions are displayed each in turn below. In each treatment page participants were given arguments with short answers below. Furthermore, participants could click the “more information” button to receive detailed information and illustrations (upon request full information can be provided). Information given in each treatment were based on information pages of the German government, governmental organization, as well as non-governmental organization. However, sources were not named to not bias participants.

Treatment 1: Benefits

Advantages for vaccinated people

For more than a year, Germany has been battling the Corona pandemic. Thanks to the Corona vaccine, a return to normalcy is finally on the horizon for vaccinated people. We have listed four of the biggest vaccination benefits for you here and would like to know whether these aspects played a role in your decision to be vaccinated or not.

1) The own protection

In short: Vaccinations protect against infection and a severe progression.

- One in ten people hospitalized for Corona require medical care
- Up to 40% of those hospitalized for corona are between 20 and 54 years old.
- One in five people hospitalized for corona dies
- Vaccinations reduce the risk of contracting Corona by 70-95%.
- Vaccinations reduce the risk of severe disease progression (hospital care) by 85%-100%.

  [> more information]

2) The protection of others

In short: Vaccinated people not only protect themselves, but others as well.

- Vaccinated people have a significantly lower risk of passing on the virus
- Vaccinated people protect all those who cannot be vaccinated for health reasons (e.g. the seriously ill, pregnant women, and children).
- The fewer infected people there are, the more the burden on the health care system is reduced
- Thus, every vaccinated person contributes to ending the pandemic

  [> more information]

3) No contact restrictions and curfews for vaccinated people

In short: Vaccinated people are exempt from contact restrictions and curfews.

- Vaccinated individuals are allowed to meet with any number of other vaccinated individuals
- They are not considered contacts
- There are no curfews for vaccinated people

  [> more information]

4) No quarantine obligation for vaccinated persons

In short: Vaccinated people are free to travel and quarantine is not required.

- More and more countries lift entry restrictions for vaccinated people
- No quarantine obligation for return travelers
- In addition, the quarantine obligation is waived after contact with infected persons

  [> more information]

Treatment 2: Debunking

Concerns about Corona vaccinations

Corona vaccinations have only recently been administered in Germany. Understandably, there are therefore many concerns among the population. We have listed four of the most common concerns here for you and would like to know whether or not the aspects mentioned play a role in your vaccination decision. Please read the information below and rate them based on their importance.

1) Is the vaccine safe despite rapid approval?

In short: Yes, thanks to prior knowledge, as well as financial and bureaucratic prioritization.

- Corona viruses have been intensively researched since 2002
- High financial support and simplification of bureaucracy
- No idle time between study phases and many volunteers

  [> more information]

2) Will a vaccine that is 70% effective protect me?

In short: Yes, it reduces the risk of infection (by 70%) and additionally the risk of a severe course. All vaccines approved in Germany reduce...

- ...the risk of infection by 70-95%.
- ...the risk of a severe course of the disease by 85-100%.

  [> more information]

3) Are the side effects already well researched?

In short: Yes, a lot of data has already been collected due to the high application.

- To detect very rare side effects, at least 100,000 applications are needed
- In Germany alone, the Corona vaccine has already been used over 30,000,000 times
- With the data collected worldwide, side effects that only occur in 1:1,000,000 people can be accurately identified.
- This far exceeds the knowledge of side effects of other medical products
- The risk of side effects is in the range of 0.0001%.

  [> more information]

4) Will the vaccine stay in my body?

In short: No, vaccinations train the immune system by having it completely break down the vaccine. Through this process, the immune system learns how to cope with an actual infection.

- Vaccines are administered only once or twice and are completely broken down within hours/days
- Therefore, they cannot accumulate in the body
- mRNA vaccines, for example, are completely degraded in less than 50 hours.
- In this degradation process, side effects may occur. However, these have already been very well researched (see point 3).
- According to the current state of knowledge, late side effects are not to be expected with the approved Corona vaccines.

  [> more information]

5C-Scale

8. **What is your attitude towards the Corona vaccines?**
   We would now like to know more about your attitude towards the vaccines. Please indicate your opinion for each of mRNA vaccines (BioNTech/Pfizer, Moderna) and vector vaccines (AstraZeneca, Johnson & Johnson). *[strongly disagree 1 |---| 7 strongly agree]*

   I have complete confidence in the safety of the vaccine.
   ... mRNA vaccines (BioNTech/Pfizer, Moderna)
   ... vector vaccines (AstraZeneca, Johnson & Johnson)

   The vaccines are effective.
   ... mRNA vaccines (BioNTech/Pfizer, Moderna)
   ... vector vaccines (AstraZeneca, Johnson & Johnson)

   As for Corona vaccines, I trust that government agencies always decide in the best interest of the public.
   ... mRNA vaccines (BioNTech/Pfizer, Moderna)
   ... vector vaccines (AstraZeneca, Johnson & Johnson)

   What is your attitude toward Corona vaccines in general?
   We would like to ask you now in more detail about Corona vaccines in general. Please indicate to what extent you agree with the following statements.

   My immune system is so strong, it also protects me from getting Corona.

   Corona is not so bad that I need to be vaccinated against it.

   Everyday stress keeps me from getting vaccinated.

   It is burdensome for me to get a vaccination.

   My discomfort with doctor visits keeps me from getting vaccinated.

   When I think about getting vaccinated, I weigh the benefits and risks to make the best decision possible.

   For each vaccination, I consider very carefully whether it makes sense for me.

   A full understanding of the vaccination issue is important to me before I get vaccinated.

   If everyone is vaccinated, I don't need to get vaccinated too.

   I get vaccinated because I can protect people with weak immune systems.

   Vaccination is a collective action to prevent the spread of disease.

Prime Check

9. **How informed do you feel?** *[not informed at all 1 |---| 7 fully informed]*
   … about the safety and effectiveness of Corona vaccines?
   … about the benefits for fully vaccinated individuals?

Intention

10. **Would you get vaccinated against Corona if you had the opportunity next week?**
    Please indicate your assessment for each of mRNA vaccines (BioNTech/Pfizer, Moderna) and vector vaccines (AstraZeneca, Johnson & Johnson).
    [Definitely would not vaccinate 1 |---| 4 Undecided |---| 7 Definitely would vaccinate]
    ... mRNA vaccines (BioNTech/Pfizer, Moderna).
    ... vector vaccines (AstraZeneca, Johnson & Johnson)

11. **Reasons Behind**
    a) If stated that either already took action and/or stated that they would be willing to get vaccinated:
    How important were / are each of the following reasons to you in your decision to get vaccinated?
    [Not important 1 |---| 7 Very important]
    ... To protect myself
    ... To protect people around me
    ... To do my part to overcome the crisis (herd immunity)
    ... To receive benefits (e.g., lifting of contact and travel restrictions)
    ... To be able to keep my job
    ... Other: ____
    ... Other: ____
    ... Other: ____

    b) If stated that did not take action yet and are not willing to get vaccinated:
    Why do you not want to be vaccinated? *[Not important 1 |---| 7 Very important]*
    ... Can't get vaccinated for medical reasons (e.g. pregnant, illness, etc.)
    ... Do not think it is necessary
    ... Think it is harmful
    ... Haven't found the time yet
    ... Don't know where to sign up...
    … Other: ____
    ... Other: ____
    ... Other: ____

Anticipated Regret

12. **Please indicate to what extent you agree with the following statements.**
    [Strongly disagree 1 |---| 7 Strongly agree]
    I am afraid that I will regret having been vaccinated if I later have side effects from the vaccination.
    I am afraid that I will regret not having been vaccinated if I later become seriously ill with corona.

Dogmatism

13. **To what extent do you agree with the following statements?** *[Do not agree at all 1 |---| 7 Fully agree]*
    Any person who honestly and truly seeks the truth will eventually come to the same conclusions as I have.
    The things I believe in are so completely true that I could never doubt them.
    My opinions are correct and will stand the test of time.
    My opinions and beliefs fit together perfectly and give a crystal clear "picture" of things.
    There are no discoveries or facts that could make me change my mind about the most important things in life.
    I am far from drawing definitive conclusions about life's central issues.
    I am absolutely certain that my ideas about the basic issues of life are correct.

If persons are "open-minded" about the most important things in life, they are likely to draw the wrong conclusions.
Twenty years from now, some of my opinions about the important things in life will probably have changed.

Vaccination denied in past

14. Have you refused vaccinations in the past or decided against vaccinating yourself or people for whom you are responsible (e.g. your child), contrary to medical advice? [*Single choice*]
    Yes
    No

Emails sent between survey experiment and follow-up survey

Participants received emails between 10 August 2021 and 30 August 2021 weekly emails informing them corresponding to their treatment. The control group was randomly split into one group receiving facilitation emails (T3) and one pure control group (C). The other two treatments (T1: Benefits and T2: Debunk) received corresponding information. Some overlap of information exist between treatments: All treatments received information on where to get the vaccination.

First email: 10 August 2021

Reminder of the

Corona Vaccination

Thank you for your participation in our spring 2021 Corona survey. This email is to remind you of the importance of Corona vaccination. We look forward to your participation in the follow-up survey in early September!

**Debunking**	**Benefits**	**Facilitation**

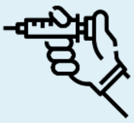

Where can I get the Corona vaccination?

For up-to-date information on making an appointment in your state, click here:

Learn more

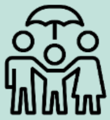

Corona vaccination from 12 years!

After extensive research, children over 12 years of age may now be vaccinated. However, there is currently an explicit recommendation only for children with pre-existing conditions.

Learn more

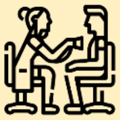

Rapid approval of the vaccines

The Corona vaccines were made available quickly and safely thanks to existing knowledge.

Learn more

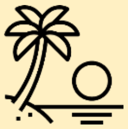

Freedom of travel for fully vaccinated

Many countries make it easier for vaccinated persons to enter the country. In addition, vaccinated persons are not required to present a negative test upon return.

Learn more

<FOOTER AS IN EMAIL 4>

Second email (16 August 2021)

Reminder of the

Corona Vaccination

Thank you for your participation in our spring 2021 Corona survey. This email is to remind you of the importance of Corona vaccination. We look forward to your participation in the follow-up survey in early September!

Debunking

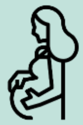

Vaccination and fertility in women

Even after more than 2 billion Corona vaccinations worldwide, there is no evidence that vaccination can affect fertility.

Learn more

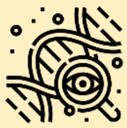

Change of the DNA?

The Corona vaccines cannot change the DNA.

Learn more

Benefits

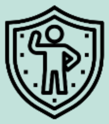

Protect yourself and others!

The Corona vaccination protects above all yourself from severe or fatal disease progression as well as long-term side effects.

Learn more

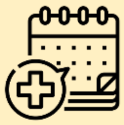

Plannability of the vaccination

In contrast to vaccination, the timing of COVID-19 infection cannot be planned and the length and intensity of the disease can be planned many times over.

Learn more

Facilitation

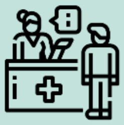

What is the vaccination procedure?

Here you will find a brief explanation of how a vaccination at the vaccination center works, how you can register and what documents are necessary.

Learn more

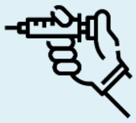

Where can I get the Corona vaccination?

For up-to-date information on making an appointment in your state, click here:

Learn more

<FOOTER AS IN EMAIL 4>

Third email (23 August 2021)

Reminder of the

Corona Vaccination

Thank you for your participation in our spring 2021 Corona survey. This email is to remind you of the importance of Corona vaccination. We look forward to your participation in the follow-up survey in early September!

Debunking

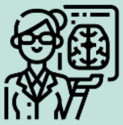

Have the side effects been adequately researched?

Vaccines have never been tested as widely as the COVID-19 vaccines. Due to the high use, the side effects could be researched exceptionally well.

Learn more

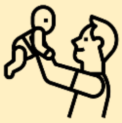

Long-term damage from vaccination?

Despite extensive vaccination, no long-term side effects have been reported to date. This and the experience of other vaccinations suggest that no long-term consequences are to be expected.

Learn more

Benefits

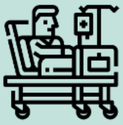

High efficacy of the vaccines

Corona vaccination prevents many Corona infections and subsequent diseases.

Learn more

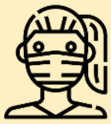

Containment of the pandemic

Vaccination protects not only vaccinated people themselves, but also others: Fully vaccinated people very rarely transmit Corona virus.

Learn more

Facilitation

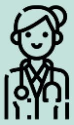

Getting vaccinated has never been easier!

There's plenty of vaccine available and it's easy to get an appointment for Corona vaccination.

Learn more

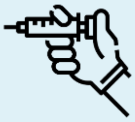

Where can I get the Corona vaccination?

For up-to-date information on making an appointment in your state, click here:

Learn more

<FOOTER AS IN EMAIL 4>

Fourth email (30 August 2021)

Reminder of the

Corona Vaccination

Thank you for your participation in our spring 2021 Corona survey. This email is to remind you of the importance of Corona vaccination. We look forward to your participation in the follow-up survey in early September!

Debunking	Benefits	Facilitation
**Vaccination concerns and fact check**<br>Many people are skeptical about the Corona vaccine. Here are the five most common concerns and what's actually true about them.<br>**Learn more**	**What are the benefits of being vaccinated?**<br>Corona vaccination offers many benefits to those vaccinated and to society. Here are seven reasons to get vaccinated.<br>**Learn more**	**Vaccination now also without appointment!**<br>In more and more vaccination centers you can get vaccinated without an appointment. Learn more at the vaccination center near you!<br>**Learn more**

Where can I get the Corona vaccination?

For up-to-date information on making an appointment in your state, click here:

Learn more

You are receiving this email because you participated in our survey on Mingle at the end of May/beginning of June and agreed to receive up to four emails. If you have any questions about this study, please feel free to contact Matthias Mayer at matthias.mayer@wiwi.uni-marburg.de.

This is a research project of the Sustainable Use of Natural Resources Working Group at the Department of Economics, Philipps-Universität Marburg.
Am Plan 1, 35037 Marburg
https://www.uni-marburg.de/en/fb02/research-groups/economics/sustuse

Philipps Universität Marburg

Icons created by monkik from www.flaticon.com
