# Supplementary figures and images for: Repeated information of benefits reduces COVID-19 vaccination hesitancy: Experimental evidence from Germany
Source: PLoS One. 2022 Jun 28;17(6):e0270666. doi: 10.1371/journal.pone.0270666 (PMC9239477; doi:10.1371/journal.pone.0270666)

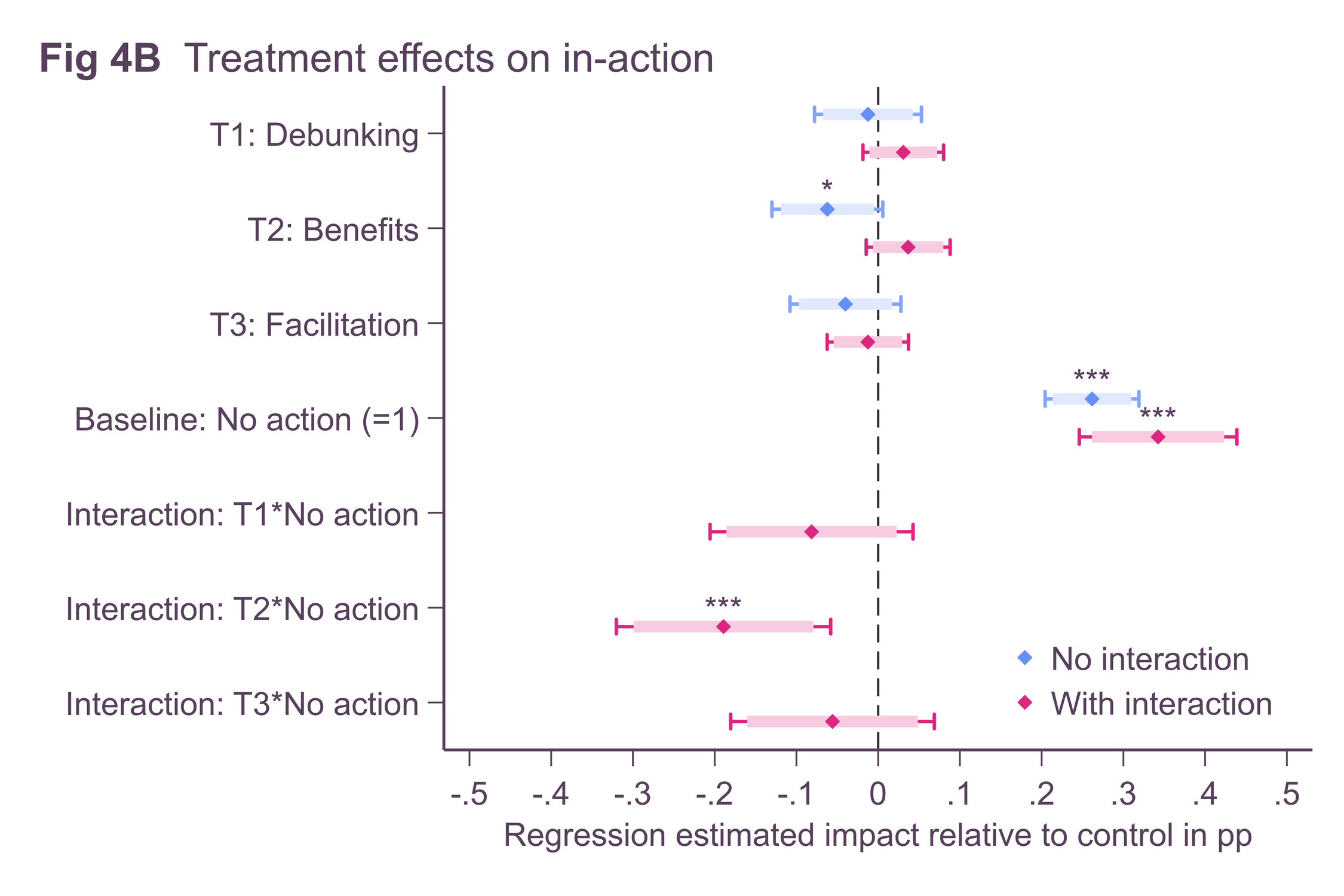

Supplement: S1 Fig — (TIF) [file pone.0270666.s013.tif]
