# Supplementary material for: Repeated information of benefits reduces COVID-19 vaccination hesitancy: Experimental evidence from Germany
Source: PLoS One. 2022 Jun 28;17(6):e0270666. doi: 10.1371/journal.pone.0270666 (PMC9239477; doi:10.1371/journal.pone.0270666)
Supplement: Attachment — Submitted filename: PONE-D-21-40313 - vaccination - review.docx [file pone.0270666.s014.docx]

**Review PLOS One - Repeated information of benefits reduce COVID-19 vaccination hesitancy: Experimental evidence from Germany - PONE-D-21-40313**

The paper is an experimental assessment using some 1,300 participants in Germany in May/June 2021, whether debunking vaccination myths, highlighting the benefits of being vaccinated, or merely sending vaccination reminders decreases hesitancy

This is simply a relevant and competent paper which gives important insights into the effectiveness of different interventions on vaccine hesitancy. The experiment is clearly explained, multiple robustness checks and checks for attrition and demand effects are conducted and presented as supplementary material. The study was pre-registered. In short: the authors know what they write about and investigate effectively and competently.

I only want to point at some minor issues:

1. Figure 3A: do you present percentages here, please make clear.
2. Is Fig3b a multiple regression, were independent variables all included at the same time?
3. Structure is a little unusual, presenting the hypotheses in the methods section. While I understand that the experiment is more clearly explained at this stage, I would advise to restructure and express hypotheses in a separate hypotheses/literature section before the methods.
4. Maybe use capital C’s when explaining 5C model for clarity.
5. On what scale was dogmatism assessed?
6. Briefly explain the platforms and software you use in one sentence, what is Respondi ? And what is ScoSci survey, is it a software?
7. Regarding your outcome measures, sometimes you talk about action, sometimes vaccination hesitancy and sometimes vaccination status. Choosing one specific term would make the text easier to read. Moreover, in measurement section, could you add the timing during which the dependent variables (intention and action) were assessed? Moreover, did you also assess vaccination intention before the treatment or only at the end of the survey? In other words, do you know whether there were differences in vaccination intention before the treatment?
8. In your sample, the older age-groups did already have the chance to get vaccinated as they were part of higher priority groups. This means that these older aged groups only consist of hesitant individuals, while the lower aged groups consist of a combination of non-hesitant and hesitant individuals. Could you conduct additional robustness checks limiting the sample to those that did not get the chance yet to get vaccinated or including interactions with age/priority groups and treatment?
9. There is much recent vaccine hesitancy material out there, also in PlosOne, also using 5C. For instance Wismans et al. (2021), Psychological characteristics and the mediating role of the 5C model in explaining students' COVID-19 vaccination intention, PlosOne, Aug 11;16(8):e0255382.
10. Avoid words like ‘now’. Imagine what that would mean if the paper is read in three year time.
