# Supplementary material for: Repeated information of benefits reduces COVID-19 vaccination hesitancy: Experimental evidence from Germany
Source: PLoS One. 2022 Jun 28;17(6):e0270666. doi: 10.1371/journal.pone.0270666 (PMC9239477; doi:10.1371/journal.pone.0270666)
Supplement: Attachment — Submitted filename: Response to Reviewers.docx [file pone.0270666.s015.docx]

Response to Reviewers on the paper:

Repeated information of benefits reduces COVID-19 vaccination hesitancy: Experimental evidence from Germany

**Response to Reviewers**

Thank you, Camelia Delcea, Reviewer 1, and Reviewer 2, for your comments provided. Some of the points raised by the reviewers echoed issues we had discussed at length prior to the initial submission of the manuscript. In these cases, it was good to get another opinion on matters where we were undecided before. In other cases, the issues raised by the reviewers made us aware of weaknesses in our manuscript we have not noticed before. In both cases, we found the feedback to be consistently constructive, well-founded, and helpful. We are very grateful for the comments provided that helped improve our manuscript.

In the following, we provide detailed responses to all comments raised by the editor and both reviewers. Changes in the manuscript based on the provided comments are included below the response in quotation marks. In case of longer fundamental changes, we only refer to the revised section in the revised manuscript with change tracking enabled.

# 1 Editor

*Journal Requirements:*

*When submitting your revision, we need you to address these additional requirements.*

*1. Please ensure that your manuscript meets PLOS ONE's style requirements, including those for file naming. The PLOS ONE style templates can be found at* 
[*https://journals.plos.org/plosone/s/file?id=wjVg/PLOSOne_formatting_sample_main_body.pdf*](https://journals.plos.org/plosone/s/file?id=wjVg/PLOSOne_formatting_sample_main_body.pdf) *and* 
[*https://journals.plos.org/plosone/s/file?id=ba62/PLOSOne_formatting_sample_title_authors_affiliations.pdf*](https://journals.plos.org/plosone/s/file?id=ba62/PLOSOne_formatting_sample_title_authors_affiliations.pdf)*.*

**Response:** We carefully checked PLOS ONE’s style requirements and revised the text, tables, and figures accordingly. The revised manuscript should now meet all these requirements.

*2. We note that you have stated that you will provide repository information for your data at acceptance. Should your manuscript be accepted for publication, we will hold it until you provide the relevant accession numbers or DOIs necessary to access your data. If you wish to make changes to your Data Availability statement, please describe these changes in your cover letter and we will update your Data Availability statement to reflect the information you provide.*

**Response:** The replication package containing the raw data and Stata code can now be accessed via GitHub (<https://github.com/IvoSteimanis/covid19_PLOS>) and Zenode (<https://zenodo.org/record/6242620>). The links were added to the corresponding fields in the submission mask.

*3. We note that Figure 2 in your submission contain [map/satellite] images which may be copyrighted. All PLOS content is published under the Creative Commons Attribution License (CC BY 4.0), which means that the manuscript, images, and Supporting Information files will be freely available online, and any third party is permitted to access, download, copy, distribute, and use these materials in any way, even commercially, with proper attribution. For these reasons, we cannot publish previously copyrighted maps or satellite images created using proprietary data, such as Google software (Google Maps, Street View, and Earth). For more information, see our copyright guidelines: http://journals.plos.org/plosone/s/licenses-and-copyright.
We require you to either (1) present written permission from the copyright holder to publish these figures specifically under the CC BY 4.0 license, or (2) remove the figures from your submission:
 
a) You may seek permission from the original copyright holder of Figure 2 to publish the content specifically under the CC BY 4.0 license.  
We recommend that you contact the original copyright holder with the Content Permission Form (http://journals.plos.org/plosone/s/file?id=7c09/content-permission-form.pdf) and the following text:
“I request permission for the open-access journal PLOS ONE to publish XXX under the Creative Commons Attribution License (CCAL) CC BY 4.0 (http://creativecommons.org/licenses/by/4.0/). Please be aware that this license allows unrestricted use and distribution, even commercially, by third parties. Please reply and provide explicit written permission to publish XXX under a CC BY license and complete the attached form.”
 Please upload the completed Content Permission Form or other proof of granted permissions as an "Other" file with your submission.
In the figure caption of the copyrighted figure, please include the following text: “Reprinted from [ref] under a CC BY license, with permission from [name of publisher], original copyright [original copyright year].”
 
b) If you are unable to obtain permission from the original copyright holder to publish these figures under the CC BY 4.0 license or if the copyright holder’s requirements are incompatible with the CC BY 4.0 license, please either i) remove the figure or ii) supply a replacement figure that complies with the CC BY 4.0 license. Please check copyright information on all replacement figures and update the figure caption with source information. If applicable, please specify in the figure caption text when a figure is similar but not identical to the original image and is therefore for illustrative purposes only.
The following resources for replacing copyrighted map figures may be helpful:
 USGS National Map Viewer (public domain): http://viewer.nationalmap.gov/viewer/
The Gateway to Astronaut Photography of Earth (public domain): http://eol.jsc.nasa.gov/sseop/clickmap/
Maps at the CIA (public domain): https://www.cia.gov/library/publications/the-world-factbook/index.html and https://www.cia.gov/library/publications/cia-maps-publications/index.html
NASA Earth Observatory (public domain): http://earthobservatory.nasa.gov/
Landsat: http://landsat.visibleearth.nasa.gov/
USGS EROS (Earth Resources Observatory and Science (EROS) Center) (public domain): http://eros.usgs.gov/#
Natural Earth (public domain): http://www.naturalearthdata.com/*

**Response**: We created the maps in Fig 2A-2B using open-source shapefiles from Open Street Map in Stata 16. We now extended the notes for Fig 2 to include all data sources and added the Open Street Map copyright to the figure. The notes now read:

“**Fig 2. Sample distribution over Germany.** Panel A shows where study participants are from. We asked participants for the first two digits of their area code and colored them according to the number of participants in each area. Panel B shows the proportion of unvaccinated individuals in Germany as of June 2, 2021. The map was created using publicly available vaccination data from the RKI [55] and shows the proportion of unvaccinated individuals by administrative code (AGS) for which the number of vaccinations was reported. For population data, we use data from the 2011 Census from the Federal Statistical Office Germany [56]. Because area codes and administrative area codes do not completely overlap, there are slight discrepancies in the areas highlighted in panels A and B. Both maps were created with Stata 16 using shapefiles from OpenStreetMap, available under the Open Database [57].”

*4. Please review your reference list to ensure that it is complete and correct. If you have cited papers that have been retracted, please include the rationale for doing so in the manuscript text, or remove these references and replace them with relevant current references. Any changes to the reference list should be mentioned in the rebuttal letter that accompanies your revised manuscript. If you need to cite a retracted article, indicate the article’s retracted status in the References list and also include a citation and full reference for the retraction notice.*

**Response:** We reviewed our reference list. To our best knowledge, none of the cited papers has been retracted. In response to the comments of the reviewers, we expanded our reference list to include the papers listed below. In the responses to the comments provided below, we address where and for what purpose the references were included.

1.	Fournet N, Mollema L, Ruijs WL, Harmsen IA, Keck F, Durand JY, et al. Under-vaccinated groups in Europe and their beliefs, attitudes and reasons for non-vaccination; two systematic reviews. BMC Public Health. 2018;18: 196. doi:10.1186/s12889-018-5103-8

2.	Altenbuchner A, Haug S, Schnell R, Scharf A, Weber K. Impfbereitschaft von Eltern mit einem COVID-19-Vakzin: Die Rolle von Elternschaft und Geschlecht. Pädiatr Pädologie. 2021;56: 230–234. doi:10.1007/s00608-021-00925-2

3.	Desson Z, Kauer L, Otten T, Peters JW, Paolucci F. Finding the way forward: COVID-19 vaccination progress in Germany, Austria and Switzerland. Health Policy Technol. 2021; 100584. doi:10.1016/j.hlpt.2021.100584

9.	Dubé E, Gagnon D, MacDonald NE. Strategies intended to address vaccine hesitancy: Review of published reviews. Vaccine. 2015;33: 4191–4203. doi:10.1016/j.vaccine.2015.04.041

10.	Dubé E, Laberge C, Guay M, Bramadat P, Roy R, Bettinger JA. Vaccine hesitancy: An overview. Hum Vaccines Immunother. 2013;9: 1763–1773. doi:10.4161/hv.24657

11.	Falagas ME, Zarkadoulia E. Factors associated with suboptimal compliance to vaccinations in children in developed countries: a systematic review. Curr Med Res Opin. 2008;24: 1719–1741. doi:10.1185/03007990802085692

12.	Larson HJ, Jarrett C, Eckersberger E, Smith DMD, Paterson P. Understanding vaccine hesitancy around vaccines and vaccination from a global perspective: A systematic review of published literature, 2007–2012. Vaccine. 2014;32: 2150–2159. doi:10.1016/j.vaccine.2014.01.081

13.	Rainey JJ, Watkins M, Ryman TK, Sandhu P, Bo A, Banerjee K. Reasons related to non-vaccination and under-vaccination of children in low and middle income countries: Findings from a systematic review of the published literature, 1999–2009. Vaccine. 2011;29: 8215–8221. doi:10.1016/j.vaccine.2011.08.096

15.	Williams SE. What are the factors that contribute to parental vaccine-hesitancy and what can we do about it? Hum Vaccines Immunother. 2014;10: 2584–2596. doi:10.4161/hv.28596

17.	Daly M, Robinson E. Willingness to Vaccinate Against COVID-19 in the U.S.: Representative Longitudinal Evidence From April to October 2020. Am J Prev Med. 2021;60: 766–773. doi:10.1016/j.amepre.2021.01.008

18.	Edwards B, Biddle N, Gray M, Sollis K. COVID-19 vaccine hesitancy and resistance: Correlates in a nationally representative longitudinal survey of the Australian population. Di Gennaro F, editor. PLOS ONE. 2021;16: e0248892. doi:10.1371/journal.pone.0248892

19.	Lazarus JV, Ratzan SC, Palayew A, Gostin LO, Larson HJ, Rabin K, et al. A global survey of potential acceptance of a COVID-19 vaccine. Nat Med. 2021;27: 225–228. doi:10.1038/s41591-020-1124-9

22.	Ruiz JB, Bell RA. Predictors of intention to vaccinate against COVID-19: Results of a nationwide survey. Vaccine. 2021;39: 1080–1086. doi:10.1016/j.vaccine.2021.01.010

23.	Troiano G, Nardi A. Vaccine hesitancy in the era of COVID-19. Public Health. 2021;194: 245–251. doi:10.1016/j.puhe.2021.02.025

24.	Cascini F, Pantovic A, Al-Ajlouni Y, Failla G, Ricciardi W. Attitudes, acceptance and hesitancy among the general population worldwide to receive the COVID-19 vaccines and their contributing factors: A systematic review. EClinicalMedicine. 2021;40: 101113. doi:10.1016/j.eclinm.2021.101113

25.	Kerr JR, Freeman ALJ, Marteau TM, van der Linden S. Effect of Information about COVID-19 Vaccine Effectiveness and Side Effects on Behavioural Intentions: Two Online Experiments. Vaccines. 2021;9: 379. doi:10.3390/vaccines9040379

26.	Lindholt MF, Jørgensen F, Bor A, Petersen MB. Public acceptance of COVID-19 vaccines: cross-national evidence on levels and individual-level predictors using observational data. BMJ Open. 2021;11: e048172. doi:10.1136/bmjopen-2020-048172

27.	Murphy J, Vallières F, Bentall RP, Shevlin M, McBride O, Hartman TK, et al. Psychological characteristics associated with COVID-19 vaccine hesitancy and resistance in Ireland and the United Kingdom. Nat Commun. 2021;12: 29. doi:10.1038/s41467-020-20226-9

30.	Gerretsen P, Kim J, Caravaggio F, Quilty L, Sanches M, Wells S, et al. Individual determinants of COVID-19 vaccine hesitancy. Inbaraj LR, editor. PLOS ONE. 2021;16: e0258462. doi:10.1371/journal.pone.0258462

31.	Wismans A, Thurik R, Baptista R, Dejardin M, Janssen F, Franken I. Psychological characteristics and the mediating role of the 5C Model in explaining students’ COVID-19 vaccination intention. Delcea C, editor. PLOS ONE. 2021;16: e0255382. doi:10.1371/journal.pone.0255382

32.	Solís Arce JS, Warren SS, Meriggi NF, Scacco A, McMurry N, Voors M, et al. COVID-19 vaccine acceptance and hesitancy in low- and middle-income countries. Nat Med. 2021;27: 1385–1394. doi:10.1038/s41591-021-01454-y

33.	Thunström L, Ashworth M, Finnoff D, Newbold SC. Hesitancy Toward a COVID-19 Vaccine. EcoHealth. 2021;18: 44–60. doi:10.1007/s10393-021-01524-0

65.	Desson Z, Lambertz L, Peters JW, Falkenbach M, Kauer L. Europe’s Covid-19 outliers: German, Austrian and Swiss policy responses during the early stages of the 2020 pandemic. Health Policy Technol. 2020;9: 405–418. doi:10.1016/j.hlpt.2020.09.003

66.	Paul KT, Zimmermann BM, Corsico P, Fiske A, Geiger S, Johnson S, et al. Anticipating hopes, fears and expectations towards COVID-19 vaccines: A qualitative interview study in seven European countries. SSM - Qual Res Health. 2022;2: 100035. doi:10.1016/j.ssmqr.2021.100035

# 2 Reviewer #1

*The paper is an experimental assessment using some 1,300 participants in Germany in May/June 2021, whether debunking vaccination myths, highlighting the benefits of being vaccinated, or merely sending vaccination reminders decreases hesitancy
This is simply a relevant and competent paper which gives important insights into the effectiveness of different interventions on vaccine hesitancy. The experiment is clearly explained, multiple robustness checks and checks for attrition and demand effects are conducted and presented as supplementary material. The study was pre-registered. In short: the authors know what they write about and investigate effectively and competently.
I only want to point at some minor issues:*

*1. Figure 3A: do you present percentages here, please make clear.*

**Response:** We agree that this was not ideally presented. We now clearly present the labels as percentages to illustrate that Fig 3A shows the percentage of respondents who took action to get vaccinated.

*2. Is Fig3b a multiple regression, were independent variables all included at the same time?*

**Response:** Yes, the plotted estimates in Fig 3B are from a multiple regression where all explanatory variables were included simultaneously, but the socio-economic controls (gender, age, education, income, marital status) are not plotted. The full model is reported in S8 Appendix (model 3). In the main text we revised the figure note to clarify this:

**“**Panel B plots regression estimates with vaccination intention (mRNA) as the dependent variable from ordinary least square regressions controlling simultaneously for the plotted variables and additionally for gender, age, education, adjusted household income, and marital status. Heteroskedasticity robust standard errors were used to compute 95% (thin bars) and 90% (thick bars) confidence intervals. Full regression outputs are reported in S9 Appendix, model 3 corresponds to the plotted estimates in panel B.**”**

*3. Structure is a little unusual, presenting the hypotheses in the methods section. While I understand that the experiment is more clearly explained at this stage, I would advise to restructure and express hypotheses in a separate hypotheses/literature section before the methods.*

**Response:** While revising the manuscript, we changed the structure several times to see what works and what does not. Ultimately, we concluded that if we mention the hypotheses in a separate Hypotheses/Literature section before the methods (as you recommended), we still need a section (similar to what we have now) at the end of the study design explaining how we answer those hypotheses with our design. This approach seems repetitive to us, as we would address the hypotheses twice. Nevertheless, we agree that the hypotheses should have been mentioned before the experimental design. The solution we have found, therefore, is to list the hypotheses in very general terms already in the introduction. In the later methods section, the hypotheses are then discussed in more detail. Moreover, and line with your comment and the comments by Reviewer 2, we decided to expand the literature in the introduction. To preserve the readability of the manuscript we decided to add an extensive literature review in the Appendix S2.

*4. Maybe use capital C’s when explaining 5C model for clarity.*

**Response:** Thank you, this is a good idea. We changed to capital C whenever we referred to any of the 5Cs in the text.

*5. On what scale was dogmatism assessed?*

**Response:** Thank you for the hint for the missed scale. We adjusted the sentence accordingly to “*Dogmatism* – the tendency to rigidly, uncritically adhere to beliefs – was assessed using a condensed version of Altemeyer’s [53] dogmatism scale, with answer options ranging from 1 “do not agree at all” to 7 “fully agree”.”

*6. Briefly explain the platforms and software you use in one sentence, what is Respondi ? And what is ScoSci survey, is it a software?*

**Response:** Respondi is the data collection firm providing access to their panel across several countries. SoSci Survey is the software used to create the survey. The server on which the survey was hosted and on which the survey was made available to participants is [www.soscisurvey.de](http://www.soscisurvey.de). We added some clarifying words highlighting the difference between the panel provider Respondi, the survey software SoSci Survey, and the server [www.soscisurvey.de](http://www.soscisurvey.de). The corresponding section now reads:

“Participants were recruited by Respondi via its online opt-in panel on the platform Mingle and directed to our surveys. In accordance with Respondi’s guidelines, participants received .75 euros per survey, each of which took on average 14 minutes to complete. Surveys were generated using the software SoSci Survey [54] and made available to participants via www.soscisurvey.de.”

*7.1 Regarding your outcome measures, sometimes you talk about action, sometimes vaccination hesitancy and sometimes vaccination status. Choosing one specific term would make the text easier to read.*

**Response:** Thank you for pointing out this inconsistency. Although we had already tried to use precise designations in the first draft, we apparently did not succeed. We have now gone through the manuscript again in detail with a focus on the named issue. We now use the term vaccination hesitancy/hesitant as a general term subsuming intentions and actions against vaccination. This general term is now only used in the introduction, discussion, and conclusion where we refer to the phenomenon in general. In the methods and results section, we use the terms vaccination intention (survey-experiment) and vaccination inaction (follow-up survey) only to refer to our specific measures.

*7.2 Moreover, in measurement section, could you add the timing during which the dependent variables (intention and action) were assessed? Moreover, did you also assess vaccination intention before the treatment or only at the end of the survey? In other words, do you know whether there were differences in vaccination intention before the treatment?*

**Response:** Both, intention to get vaccinated and the 5C scale were only asked after the treatment to avoid demand effects. We chose this between-subject design as we were concerned that participants might draw conclusions about the background of the study by eliciting intention to get vaccination both before and after treatment, which in turn might have influenced their response behavior. As suggested, we added a clarifying sentence to the paragraphs in the methods section that explain intention and action, respectively:

*Intention:* “Both intention to get vaccinated and the 5C scale were measured after the treatment to avoid demand effects.”

*Action:* “Vaccination action was assessed prior to the treatment in the survey-experiment.”

Since we did not ask participants regarding their intention prior to the treatment we cannot rule out the possibility that differences between groups regarding the intention existed. However, to keep the chances of that happening low, participants were assigned randomly to one of the four treatment groups. There was no statistically significant difference between reported vaccination in-action between groups (see balancing test in Appendix S7, Table S5).

*8. In your sample, the older age-groups did already have the chance to get vaccinated as they were part of higher priority groups. This means that these older aged groups only consist of hesitant individuals, while the lower aged groups consist of a combination of non-hesitant and hesitant individuals. Could you conduct additional robustness checks limiting the sample to those that did not get the chance yet to get vaccinated or including interactions with age/priority groups and treatment?*

**Response:** Thank you for this suggestion. We do not find that unvaccinated participants with priority status are systematically more hesitant than the rest of our sample. We included the corresponding analysis in S9 Appendix, which reads as follows:

“We thank an anonymous reviewer for the suggestion to investigate heterogeneous treatment effects depending on participants' priority group status. In Germany, vaccination priorities were ranked by age, medical condition, and profession (health care, nursing, etc.). At the time of the survey experiment, prioritization was lifted and all people above the age of 16 could apply for a vaccination appointment at vaccination centers or their general practitioner. Officially, vaccination prioritization ended in Germany on June 7th, but because of the high demand, appointments were organized several weeks in advance. In our sample, 33 participants (2.5%) reported to be in the first prioritization group (highest priority), 129 (9.7%) in the second priority group (high priority), and 342 (25,8%) in the third group (increased priority). Thus, about 38% of participants in the survey experiment could potentially be more hesitant than the rest, because they already had the chance to get vaccinated, or at least to get an appointment, due to their priority status.

First, looking at how vaccination intentions vary across priority groups (see Fig S6A), we find that intentions are indeed slightly lower for participants in group 1 (the highest priority group) compared to participants without priority status (T-Test diff. = -.93, t_851_ = -2.15, p = .03). Importantly, participants in group 2 (high priority) have similar intentions (T-Test diff. = .09, t_947_ = .41, p = .68) and participants in group 3 (increased priority) even have significant higher intentions compared to participants without priority status (T-Test diff. = 1.42, t_1,160_ = 9.96, p < .001). Second, we analyze whether participants respond differently to the treatments in the survey experiment depending on their priority status (see Fig S6B). Given the low number of observations in each treatment condition of participants in the first two priority groups, we opted for a binary specification of priority status (which equals 1 if a respondent is in priority group 1, 2, or 3 and equals 0 if a respondent has no priority status). We find that among the group of participants with priority status, intentions to get vaccinated are slightly higher in the benefits treatment compared to the control group (ß = .37, p = .07, 95%CI: -.03 to .77). In addition, we find that none of the treatments significantly affected vaccination intentions among participants with priority status. This could be seen as suggestive evidence that the benefits treatment increased vaccination intentions only among participants without priority status (but only at the 10% significance level).

Fig. S1. Vaccination intentions

**Fig. S6.	Vaccination intentions**. Panel A shows average intentions to get vaccinated with an mRNA vaccine across priority groups. Panel B shows treatment effects by priority status with a binary specification that equals 1 if the participant reported being in one of the three priority groups and 0 otherwise. Dashed lines indicate 95% confidence intervals.

Taken together, these findings speak against our prior belief that unvaccinated participants with priority status are systematically more hesitant than the rest of the sample. It might be the case that participants with priority group status still faced barriers in getting their vaccination. For example, they might have struggled to make an appointment which often required either computer skills or waiting in long phone queues. Especially older participants, who predominantly made up the highest priority groups, might have struggled with this. In the survey experiment, 70% of participants with priority status reported that they have a vaccination appointment (27%) or are on a waiting list (43%). Thus, it seems that most participants with priority status in our sample only have or had to delay their vaccination but were still getting their vaccination faster than participants without the priority status (only 8% of them had an appointment and 26% were on a waiting list).”

*9. There is much recent vaccine hesitancy material out there, also in PlosOne, also using 5C. For instance Wismans et al. (2021), Psychological characteristics and the mediating role of the 5C model in explaining students' COVID-19 vaccination intention, PlosOne, Aug 11;16(8):e0255382.*

**Response**: In line with your comment and the comments 1-3 of reviewer 2 we now elaborate more on psychological determinants of hesitancy in general and the 5Cs in relation to COVID-19 in particular. We tried to keep our introduction as short as possible and only included a highly condensed version of the literature review. However, – in line with reviewers 2 suggestion – a more extensive literature review is provided in S2 Appendix.

*10. Avoid words like ‘now’. Imagine what that would mean if the paper is read in three year time*

**Response:** Yes, good point. We adjusted the respective passages accordingly.

“The COVID-19 pandemic prompted global research leading to the development of several vaccines in record time. At the same time, countries like Germany are ~~now~~ struggling to have enough people vaccinated to relieve pressure on their healthcare systems.”

“Meanwhile, vaccine protection is waning [5,6], and new variants are ~~the emerging Omicron variant is~~ expected to lead to further ~~another~~ waves of infections that could bring public life ~~once~~ to a standstill again [7].“

# 3 Reviewer #2

*Perhaps the chief concern I have with the paper – given its pre-registration, careful design and statistical analysis – and nuanced and transparent analysis of data – is the motivation articulated for the research and the specific interventions.*

*I know your interventions are also pre-registered and I will not suggest you change those or the hypotheses posed – they are what they are. But I do think you need to do a better job in outlining “state of the art” regarding (a) what we know about vaccine hesitancy in general, (b) what we know about vaccine hesitancy in relations to Covid-vaccination, and (c) also expand on what is known in the political-cultural-institutional setting of this research (Germany, or perhaps German-speaking countries in general, which you argue your results to generalize to). In the below I briefly expand on each of these pointers and why I see revisions needed:*

*1. Regarding (a) what we know about vaccine hesitancy in general. You cite two of the classic recent overview articles by Dubé and Schmid but you do not provide any generic overview of what is known in general population samples to explain large sources of variation in vaccine uptake or self-reported measures of vaccine hesitancy. I think this should be the basic starting point for any research on the topic. Since there are meta-analyses and literature reviews readily available, condensing that information into a new appendix (summarized shortly in the introduction) or a few sentences of new text in the introduction seems warranted.*

**Response:** Indeed, prior to our initial submission, we had lengthy discussions about whether to include a review of the literature on general vaccine hesitancy. In our first draft, we ultimately decided against it. However, your comment confirmed our initial hunch to include such a general literature review as an introduction to the topic. We therefore expanded the literature on the determinants of overall vaccination hesitancy in the introduction. We added a highly condensed summary of the literature in the introduction and a more extensive review to S2 Appendix, discussing (a) general vaccination hesitancy, (b) vaccination hesitancy in relation to COVID-19, and (c) vaccination hesitancy in relation to COVID-19 in Germany. The introduction now reads as follows:

“Vaccination hesitancy – the delay in accepting or refusing vaccines despite their availability [8] – has been extensively researched prior to COVID-19. Decades of vaccination hesitancy research has shown that hesitancy is complex, multifaceted, and context specific (for an overview see [9]). A large share of variation in hesitancy can be explained by differences in (i) sociodemographic characteristics; (ii) cultural, institutional, and political factors; and (iii) psychological factors [10–15]. However, there is not one universal factor that consistently explains hesitancy as determinants vary not only across countries and vaccine types but also over time."

References added to the main text regarding general vaccination hesitancy are:

9.	Dubé E, Gagnon D, MacDonald NE. Strategies intended to address vaccine hesitancy: Review of published reviews. Vaccine. 2015;33: 4191–4203. doi:10.1016/j.vaccine.2015.04.041

10.	Dubé E, Laberge C, Guay M, Bramadat P, Roy R, Bettinger JA. Vaccine hesitancy: An overview. Hum Vaccines Immunother. 2013;9: 1763–1773. doi:10.4161/hv.24657

11.	Falagas ME, Zarkadoulia E. Factors associated with suboptimal compliance to vaccinations in children in developed countries: a systematic review. Curr Med Res Opin. 2008;24: 1719–1741. doi:10.1185/03007990802085692

12.	Larson HJ, Jarrett C, Eckersberger E, Smith DMD, Paterson P. Understanding vaccine hesitancy around vaccines and vaccination from a global perspective: A systematic review of published literature, 2007–2012. Vaccine. 2014;32: 2150–2159. doi:10.1016/j.vaccine.2014.01.081

13.	Rainey JJ, Watkins M, Ryman TK, Sandhu P, Bo A, Banerjee K. Reasons related to non-vaccination and under-vaccination of children in low and middle income countries: Findings from a systematic review of the published literature, 1999–2009. Vaccine. 2011;29: 8215–8221. doi:10.1016/j.vaccine.2011.08.096

15.	Williams SE. What are the factors that contribute to parental vaccine-hesitancy and what can we do about it? Hum Vaccines Immunother. 2014;10: 2584–2596. doi:10.4161/hv.28596

*2. Regarding what we know about vaccine hesitancy in relations to Covid-vaccination, your current outline merely focus on sociodemographics such as age, gender, and education (Edwards et al., 2021; Neumann-Böhme et al., 2020; Petersen et al., 2021; Ruiz & Bell, 2021). There are of course 100s if not more studies already available, and I certainly agree you cannot summarize all of these, but I am still somewhat confused about the focus solely on these sociodemographics, and why? (are they more generalizeable across countries? Do they explain more variation in general population samples, etc). A further motivation why solely summarizing current knowledge as consisting of these -and not other – factors is needed. I also think you should do a further cursory review looking specifically at large-N (many thousands) studies of general population samples that you judge are high quality studies, and considering including a few other central factors if you deem them relevant. I have no personal pet peeve here, but I am concerned about carefully summarizing “known knowns” and motivating variable foci.*

**Response:** Yes, we agree. It was not our intention to put the focus on socio-economics. Socio-economic factors are indeed not at the core of our analysis but rather peripheral as the intervention in our study aims at altering psychological factors, i.e., increasing beliefs of safety and benefits of the vaccine and reduction of constraints. Therefore, the comment on your confusion regarding our focus is well-received. We now shifted the focus in the parts reviewing the literature away from socio-economics and towards psychological factors. In line with comment 9 of Reviewer 1, we also added further literature on the 5Cs in relation to COVID-19 vaccination hesitancy. The new section now reads:

”Research on hesitancy towards COVID-19 vaccines shed further light on the connection of the three broader categories researched in vaccination hesitancy in general. First, sociodemographic characteristic such as age, gender, affluency, and education were associated with vaccination hesitancy in many different contexts [16–24]. Second, cultural, political, and institutional differences such as lower trust in authorities and support of populist views were also found to correlate with higher COVID-19 hesitancy [18,24–27]. Third, psychological factors such as higher risk appraisal, greater psychological distress from the pandemic, and stronger other regarding preferences have been consistently shown to correlate with lower hesitancy [27–31].

While sociodemographic characteristics as well as cultural, institutional, and political factors are important to explain how hesitancy emerges, it is difficult, sometimes even impossible, to change them. Policies targeting people’s attitudes, perceptions, and opinions are likely more fruitful in reducing hesitancy quickly. Moreover, doubts regarding the safety, effectiveness, and benefits of the COVID-19 vaccines on the one hand and an underestimation of the risk of infection and severe illness on the other have been shown to be the most prominent reasons for hesitancy across many countries [24,26,28,29,32,33]. One approach to mapping these psychological determinants is the 5C model, which elicits people’s Confidence in the safety and efficacy of the vaccine, Complacency about the risk of infection, Constraints that prevent one from vaccinating, Calculation of one’s own costs and benefits, and the perceived Collective responsibility to vaccinate [34]. Furthermore, analysis suggests that many psychological factors determining hesitancy towards COVID-19 vaccines are mediated via the 5Cs [31].”

The literature added to this section is:

17.	Daly M, Robinson E. Willingness to Vaccinate Against COVID-19 in the U.S.: Representative Longitudinal Evidence From April to October 2020. Am J Prev Med. 2021;60: 766–773. doi:10.1016/j.amepre.2021.01.008

18.	Edwards B, Biddle N, Gray M, Sollis K. COVID-19 vaccine hesitancy and resistance: Correlates in a nationally representative longitudinal survey of the Australian population. Di Gennaro F, editor. PLOS ONE. 2021;16: e0248892. doi:10.1371/journal.pone.0248892

19.	Lazarus JV, Ratzan SC, Palayew A, Gostin LO, Larson HJ, Rabin K, et al. A global survey of potential acceptance of a COVID-19 vaccine. Nat Med. 2021;27: 225–228. doi:10.1038/s41591-020-1124-9

22.	Ruiz JB, Bell RA. Predictors of intention to vaccinate against COVID-19: Results of a nationwide survey. Vaccine. 2021;39: 1080–1086. doi:10.1016/j.vaccine.2021.01.010

23.	Troiano G, Nardi A. Vaccine hesitancy in the era of COVID-19. Public Health. 2021;194: 245–251. doi:10.1016/j.puhe.2021.02.025

24.	Cascini F, Pantovic A, Al-Ajlouni Y, Failla G, Ricciardi W. Attitudes, acceptance and hesitancy among the general population worldwide to receive the COVID-19 vaccines and their contributing factors: A systematic review. EClinicalMedicine. 2021;40: 101113. doi:10.1016/j.eclinm.2021.101113

25.	Kerr JR, Freeman ALJ, Marteau TM, van der Linden S. Effect of Information about COVID-19 Vaccine Effectiveness and Side Effects on Behavioural Intentions: Two Online Experiments. Vaccines. 2021;9: 379. doi:10.3390/vaccines9040379

26.	Lindholt MF, Jørgensen F, Bor A, Petersen MB. Public acceptance of COVID-19 vaccines: cross-national evidence on levels and individual-level predictors using observational data. BMJ Open. 2021;11: e048172. doi:10.1136/bmjopen-2020-048172

27.	Murphy J, Vallières F, Bentall RP, Shevlin M, McBride O, Hartman TK, et al. Psychological characteristics associated with COVID-19 vaccine hesitancy and resistance in Ireland and the United Kingdom. Nat Commun. 2021;12: 29. doi:10.1038/s41467-020-20226-9

30.	Gerretsen P, Kim J, Caravaggio F, Quilty L, Sanches M, Wells S, et al. Individual determinants of COVID-19 vaccine hesitancy. Inbaraj LR, editor. PLOS ONE. 2021;16: e0258462. doi:10.1371/journal.pone.0258462

31.	Wismans A, Thurik R, Baptista R, Dejardin M, Janssen F, Franken I. Psychological characteristics and the mediating role of the 5C Model in explaining students’ COVID-19 vaccination intention. Delcea C, editor. PLOS ONE. 2021;16: e0255382. doi:10.1371/journal.pone.0255382

32.	Solís Arce JS, Warren SS, Meriggi NF, Scacco A, McMurry N, Voors M, et al. COVID-19 vaccine acceptance and hesitancy in low- and middle-income countries. Nat Med. 2021;27: 1385–1394. doi:10.1038/s41591-021-01454-y

33.	Thunström L, Ashworth M, Finnoff D, Newbold SC. Hesitancy Toward a COVID-19 Vaccine. EcoHealth. 2021;18: 44–60. doi:10.1007/s10393-021-01524-0

*3. Regarding what is known about vaccine hesitancy in relations in the political-cultural-institutional setting of this research (Germany, or German-speaking countries in general), some discussion is needed that (i) vaccine hesitancy is parly demographic (education, age, gender, etc (Schwarzinger et al., 2021; Troiano & Nardi, 2021)) partly psychological (perceptions, fear, personality types, etc (Gerretsen et al., 2021; Murphy et al., 2021; Wismans et al., 2021)) and also (interacting with both demographics and psychology) political-cultural-institutional in its determinants. For example, we know that adherents to specific religious beliefs, though traditions (in your context e.g. The Rudolf-Steiner tradition of Anthroposophy (Fournet et al., 2018)) and political (e.g. right-wing voters in the US and perhaps also in Europe (Ruiz & Bell, 2021)) ideologies express significant differences in vaccination intention and I would guess, also in vaccination uptake. I thus think you need to somehow mention that each country-culture may face rather unique challenges in vaccine uptake, for political-cultural-institutional reasons (in many countries also due to lack of medical infrastructure) and thus contextualize your research better. When you have done so, zooming in on what may be specific in the German-speaking context (there are general population surveys available such as (Graeber et al., 2021) would be proper contextualization of the current study, its design and interventions, and also in discussing the results.*

**Response:** Additionally to the literature on (a) general vaccination hesitancy and (b) COVID-19 specific vaccionation hesitancy we now also added a sentence on (c) the situation of COVID-19 vaccination hesitancy in Germany. As we did not intend to investigate the impact of identification with specific ideologies – such as anthroposophy, homeopathy, or right-wing support – we did not consider these further in our data collection or analysis. Since we cannot make claims regarding this point with our own data we decided to mention these factors potentially benefiting vaccination hesitancy in Germany only in passing. The added section in the introduction reads:

At the same time, countries like Germany are struggling to relieve pressure on their healthcare systems by getting as many people vaccinated as possible. “The prevalence of certain ideologies in Germany – such as anthroposophy [1], homeopathy [2], and far right-wing supporters [3] – likely provided fertile grounds for skepticism towards COVID-19 vaccines.”

The literature added to this aspect is:

1.	Fournet N, Mollema L, Ruijs WL, Harmsen IA, Keck F, Durand JY, et al. Under-vaccinated groups in Europe and their beliefs, attitudes and reasons for non-vaccination; two systematic reviews. BMC Public Health. 2018;18: 196. doi:10.1186/s12889-018-5103-8

2.	Altenbuchner A, Haug S, Schnell R, Scharf A, Weber K. Impfbereitschaft von Eltern mit einem COVID-19-Vakzin: Die Rolle von Elternschaft und Geschlecht. Pädiatr Pädologie. 2021;56: 230–234. doi:10.1007/s00608-021-00925-2

3.	Desson Z, Kauer L, Otten T, Peters JW, Paolucci F. Finding the way forward: COVID-19 vaccination progress in Germany, Austria and Switzerland. Health Policy Technol. 2021; 100584. doi:10.1016/j.hlpt.2021.100584

Furthermore, we highlight the similarities between Germany and two other German-speaking countries (Austria and Switzerland) in the subsection “Limitaitons” of the section “Discussion”. Here we refer to four recent studies highlighting similarities in their political, cultural, and institutional characteristics, early responses to COVID-19, and plateauing uptake rates in September 2021.

“Our results might thus be more transferable to countries with similar contexts, such as Austria and Switzerland. These German-speaking countries have substantial cultural, historical, and economic ties and share a similar system of federalism with mandatory universal health insurance [3,65,66]. While all three countries had prompt and similar governmental responses to the COVID-19 outbreak (school closures, obligatory masks, enforce social distancing, free testing facilities, contact tracing, etc.) vaccination rates plateaued by the end of September 2021 despite readily available vaccines [3].”

The literature added to this aspect is:

65.	Desson Z, Lambertz L, Peters JW, Falkenbach M, Kauer L. Europe’s Covid-19 outliers: German, Austrian and Swiss policy responses during the early stages of the 2020 pandemic. Health Policy Technol. 2020;9: 405–418. doi:10.1016/j.hlpt.2020.09.003

66.	Paul KT, Zimmermann BM, Corsico P, Fiske A, Geiger S, Johnson S, et al. Anticipating hopes, fears and expectations towards COVID-19 vaccines: A qualitative interview study in seven European countries. SSM - Qual Res Health. 2022;2: 100035. doi:10.1016/j.ssmqr.2021.100035

In the case of Germany-specific literature on factors associated with vaccination hesitancy, a more conclusive literature review has been added to Appendix S2 subsection Vaccination hesitancy in Germany.

*I have carefully read the methods and analyses and have little to complain on. There is a large attrition cross survey waves but it seems to be random, and you handle it well. The statistical analyses are careful and transparent, and also nicely summarized graphically. I especially like the post-hoc tests and the nice graphical summarize of self-reported reasons in Figure 6. Also figure 7 – in summarizing potentially more useful interventions – is very illuminating. Well done!*

*One minor reservetation though is your dismissal of the rather established 5Cs model. You write that “Fig. 5 shows the effect each item of the 5Cs measured in the first wave has on self-reported vaccination behavior. Interestingly, the model controlling for other reasons such covid risk perception, emotional response, dogmatism, and anticipated regret has the same explanatory power (adjusted R2=0.35) indicating that these factors are already captured by the 5Cs”. I do not agree. Different covariates may explain equal / similar share of variance, even if they do not overlap much in the covariance matrix (the hold out predictors will just be in the residual). To firmly draw the conclusions that your variables covid risk perception, emotional response, dogmatism, and anticipated regret are already captured by the 5Cs variables, I would suggest a mediation analysis. Obviously, they are correlated, but I do not understand how you draw the conclusion they are “already captured” by the 5C variables?*

**Response:** Thank you for the helpful comment regarding the 5C model. We think this could be due to our imprecise writing and overselling the point we wanted to make about the importance of the 5Cs in predicting vaccination behavior.

We used a stepwise modeling approach, starting from a minimal model with only the treatments (model 1 in table 1 below), and sequentially added sets of explanatory variables to test which models best predict hesitancy in our sample. The model controlling for other reasons (model 4) explains about 25% of the unique variance in hesitancy (adj. R^2^ = .25), with net anticipated regret appearing to be a strong predictor. But controlling simultaneously for the 5C and these other reasons does not further increase the share of explained variation and the estimate of net anticipated regret turns insignificant - which is why we concluded that the 5C already ‘capture’ these other reasons and seem to be more powerful predictors of vaccine hesitancy. A general to specific modeling algorithm (‘gets’, model 6) fed with socio-economic, 5Cs, and the other reasons keeps only three of the 5C factors (Confidence mRNA, Calculation, and Collective) as independent variables which already explain 34% of the unique variation (adj. R^2^ = .34).

Table 1 Determinants of inaction

|  | **Inaction (=1)** | | | | | |
| --- | --- | --- | --- | --- | --- | --- |
| VARIABLES | (1) | (2) | (3) | (4) | (5) | GETS  (6) |
|  |  |  |  |  |  |  |
| T1: Debunking (=1) | -0.05 | -0.05 | -0.06 | -0.05 | -0.06 |  |
|  | (0.07) | (0.07) | (0.06) | (0.06) | (0.06) |  |
| T2: Benefits (=1) | -0.16** | -0.18** | -0.13** | -0.15** | -0.13** |  |
|  | (0.07) | (0.07) | (0.06) | (0.06) | (0.06) |  |
| T3: Facilitator (=1) | -0.08 | -0.09 | -0.08 | -0.06 | -0.08 |  |
|  | (0.07) | (0.07) | (0.06) | (0.06) | (0.06) |  |
| ***Socio-economics:*** |  |  |  |  |  |  |
| Female (=1) |  | 0.12** | 0.03 | 0.04 | 0.03 |  |
|  |  | (0.05) | (0.04) | (0.04) | (0.04) |  |
| Age: 30-39 (=1) |  | -0.05 | -0.21** | -0.15 | -0.22** |  |
|  |  | (0.10) | (0.09) | (0.10) | (0.10) |  |
| Age: 40-49 (=1) |  | -0.07 | -0.16** | -0.11 | -0.16** |  |
|  |  | (0.09) | (0.08) | (0.08) | (0.08) |  |
| Age: 50-64 (=1) |  | -0.03 | -0.14* | -0.09 | -0.15* |  |
|  |  | (0.08) | (0.08) | (0.08) | (0.08) |  |
| Age: 65+ (=1) |  | 0.08 | -0.13 | -0.07 | -0.14 |  |
|  |  | (0.10) | (0.09) | (0.10) | (0.10) |  |
| Secondary school (=1) |  | -0.04 | -0.02 | -0.01 | -0.01 |  |
|  |  | (0.08) | (0.06) | (0.07) | (0.06) |  |
| High school (=1) |  | -0.10 | -0.01 | 0.01 | 0.01 |  |
|  |  | (0.08) | (0.07) | (0.07) | (0.07) |  |
| University degree (=1) |  | -0.15* | -0.06 | -0.04 | -0.05 |  |
|  |  | (0.09) | (0.07) | (0.07) | (0.07) |  |
| Adjusted HH income |  | -0.02 | -0.01 | -0.02 | -0.01 |  |
|  |  | (0.01) | (0.01) | (0.01) | (0.01) |  |
| Married (=1) |  | 0.03 | 0.00 | 0.00 | -0.00 |  |
|  |  | (0.05) | (0.04) | (0.05) | (0.04) |  |
| ***Baseline: Five C*** |  |  |  |  |  |  |
| Confidence: MRNA |  |  | -0.10*** |  | -0.10*** | -0.08*** |
|  |  |  | (0.02) |  | (0.02) | (0.02) |
| Confidence: VECTOR |  |  | 0.01 |  | 0.01 |  |
|  |  |  | (0.03) |  | (0.03) |  |
| Complacency |  |  | -0.03* |  | -0.03* |  |
|  |  |  | (0.01) |  | (0.02) |  |
| Constraints |  |  | 0.01 |  | 0.01 |  |
|  |  |  | (0.02) |  | (0.02) |  |
| Calculation |  |  | 0.04*** |  | 0.04** | 0.03** |
|  |  |  | (0.02) |  | (0.02) | (0.01) |
| Collective |  |  | -0.07*** |  | -0.07*** | -0.06*** |
|  |  |  | (0.02) |  | (0.02) | (0.02) |
| ***Baseline: Other Reasons*** |  |  |  |  |  |  |
| Denied other vaccine (=1) |  |  |  | 0.05 | -0.06 |  |
|  |  |  |  | (0.06) | (0.06) |  |
| Index: Covid risk perception std.) |  |  |  | -0.01 | 0.00 |  |
|  |  |  |  | (0.03) | (0.03) |  |
| Index: Emotional response (std.) |  |  |  | -0.00 | 0.00 |  |
|  |  |  |  | (0.03) | (0.03) |  |
| Net anticipated regret (std.) |  |  |  | -0.24*** | -0.02 |  |
|  |  |  |  | (0.02) | (0.04) |  |
| Index: Dogmatism (std.) |  |  |  | 0.03 | 0.03 |  |
|  |  |  |  | (0.02) | (0.02) |  |
| Constant | 0.58*** | 0.68*** | 1.23*** | 0.58*** | 1.25*** | 0.89*** |
|  | (0.05) | (0.12) | (0.16) | (0.11) | (0.17) | (0.10) |
|  |  |  |  |  |  |  |
| Observations | 441 | 441 | 441 | 441 | 441 | 441 |
| R-squared | 0.01 | 0.06 | 0.38 | 0.28 | 0.38 | 0.34 |
| Adjusted R-squared | 0.007 | 0.027 | 0.352 | 0.250 | 0.348 | 0.339 |

Notes: The table shows estimates from linear probability models (1 to 5) with inaction as the outcome variable for the subsample of participants that had not taken any actions in the survey experiment. Model 6 shows the results from a GETS model. Heteroskedasticity robust standard errors in parentheses (*** p < .01, ** p < .05, * p < .1).

We revised this section and toned down the language and added models 4 and 6 from table 1 above to S9 Appendix in the revised version.

The section now reads

“Using a stepwise modeling approach shows that socio-economic characteristics are jointly significant predictors of vaccination behavior, but they only explain 3% of the unique variation (adj. R2 = .03). Additionally controlling for the 5Cs increases the explained variation by more than tenfold (adj. R2 = .35). The model controlling for other reasons such as COVID-19 risk perception, emotional response, dogmatism, and anticipated regret in addition socioeconomics explain about 25% of the variation (adj. R2 = .25). However, when all factors are simultaneously controlled for, the model has the same explanatory power (adj. R2 = .35) as the model only controlling for socioeconomics and the 5Cs – indicating the importance of the 5Cs (S9 Appendix).”
