# Supplementary material for: Repeated information of benefits reduces COVID-19 vaccination hesitancy: Experimental evidence from Germany
Source: PLoS One. 2022 Jun 28;17(6):e0270666. doi: 10.1371/journal.pone.0270666 (PMC9239477; doi:10.1371/journal.pone.0270666)
Supplement: Attachment — Submitted filename: 02_Response to Reviewers.docx [file pone.0270666.s016.docx]

Response to Reviewers on the paper:

Repeated information of benefits reduces COVID-19 vaccination hesitancy: Experimental evidence from Germany

**Response to Reviewers**

# 1 Reviewer #1

*Dear authors,*

*you dealt with all the comments.*

*The only minor thing missing may be a robustness check without the priority groups. you could with its results in a footnote.*

*good luck.*

*you did a fine job*

**Response:** Dear reviewer, thank you for your kind comments and helpful recommendations. We adapted the manuscript in the suggested manner. However, since PLOS ONE does not allow for footnotes, we included the suggested text in the main manuscript reading:

“One concern could be that respondents belonging to a priority group are more hesitant than other respondents as they had more time to get vaccinated but are not. Testing for this possibility we find no differences in treatment effects between priority and non-priority groups. Furthermore, respondents belonging to a priority group reported higher intentions than respondents without priority status, not lower. Beyond, we preregistered several heterogeneous effects but do not find any of them to be statistically significant ~~(see S9 Appendix)~~. In all estimations, we additionally control for differences in covariates between treatment groups (see S9 Appendix for additional results and robustness checks).”

Furthermore, Table S11 displaying the regression of the subsample of respondents without priorization status was added to Appendix S9.

Table S11. Treatment effects vaccination intentions: Only respondents without prioritization status

|  | mRNA | | |
| --- | --- | --- | --- |
| VARIABLES | (1) | (2) | (3) |
|  |  |  |  |
| T1: Debunk | 0.15 | 0.11 | 0.03 |
|  | (0.21) | (0.21) | (0.13) |
| T2: Benefits | 0.37* | 0.30 | 0.19 |
|  | (0.20) | (0.20) | (0.12) |
| ***Socio-economics*** |  |  |  |
| Female (=1) |  | -0.23 | -0.16 |
|  |  | (0.17) | (0.11) |
| Age: 32-44 (=1) |  | -0.49* | 0.03 |
|  |  | (0.29) | (0.21) |
| Age: 45-52 (=1) |  | -0.35 | -0.25 |
|  |  | (0.26) | (0.16) |
| Age: 53-58 (=1) |  | -0.52** | -0.29* |
|  |  | (0.24) | (0.16) |
| Age: 59-81 (=1) |  | -1.97*** | -0.78*** |
|  |  | (0.42) | (0.27) |
| Secondary school: ‘Realschulabschluss’ (=1) |  | 0.48 | 0.17 |
|  |  | (0.31) | (0.20) |
| High school (=1) |  | 0.80** | 0.18 |
|  |  | (0.32) | (0.21) |
| University degree (=1) |  | 0.93*** | 0.07 |
|  |  | (0.32) | (0.21) |
| Adjusted HH income |  | 0.05 | -0.02 |
|  |  | (0.05) | (0.03) |
| Married (=1) |  | -0.10 | -0.17 |
|  |  | (0.19) | (0.12) |
| ***Reasons*** |  |  |  |
| Vaccination inaction (=1) |  |  | -0.92*** |
|  |  |  | (0.12) |
| Denied other vaccine (=1) |  |  | -0.55*** |
|  |  |  | (0.18) |
| Index: COVID-19 risk perception std.) |  |  | 0.23*** |
|  |  |  | (0.07) |
| Index: Emotional response (std.) |  |  | 0.08 |
|  |  |  | (0.07) |
| Net anticipated regret (std.) |  |  | 1.40*** |
|  |  |  | (0.06) |
| Index: Dogmatism (std.) |  |  | -0.03 |
|  |  |  | (0.05) |
| Constant | 4.68*** | 4.48*** | 5.90*** |
|  | (0.12) | (0.37) | (0.25) |
|  |  |  |  |
| Observations | 820 | 820 | 820 |
| R-squared | 0.00 | 0.06 | 0.64 |
| Adjusted R-squared | 0.002 | 0.048 | 0.628 |
| F-test: Socio-economics |  | 0.000 | 0.007 |
| F-test: Reasons |  |  | 0.000 |

Notes: The dependent variable is the intention to get vaccinated, separately for mRNA and vector vaccines, measured using a 7-point Likert scale. Estimates are obtained from multiple least square regressions with robust standard errors in parentheses: *** p<.01, ** p<.05, * p<.1.
